# MTFR2‐Mediated Fission Drives Fatty Acid and Mitochondrial Co‐Transfer from Hepatic Stellate Cells to Tumor Cells Fueling Oncogenesis

**DOI:** 10.1002/advs.202416419

**Published:** 2025-05-14

**Authors:** La Zhang, Baoyong Zhou, Jun Yang, Cong Ren, Jing Luo, Zhenghang Li, Qiang Liu, Zuotian Huang, Zhongjun Wu, Ning Jiang

**Affiliations:** ^1^ Department of Hepatobiliary Surgery The First Affiliated Hospital of Chongqing Medical University, College of Basic Medical Sciences of Chongqing Medical University Chongqing 400016 China; ^2^ Department of Pathology, College of Basic Medical Sciences Chongqing Medical University Chongqing 400016 China; ^3^ Molecular Medicine Diagnostic and Testing Center Chongqing Medical University Chongqing 400016 China; ^4^ Department of Pathology The First Affiliated Hospital of Chongqing Medical University Chongqing 400016 China; ^5^ Department of Hepatobiliary Surgery Bishan Hospital of Chongqing Medical University Chongqing 400016 China; ^6^ Department of Anesthesiology The First Affiliated Hospital of Chongqing Medical University Chongqing 400016 China; ^7^ Department of Medicinal Chemistry College of Pharmacy Chongqing Medical University Chongqing 400016 China

**Keywords:** fatty acid transfer, hepatic stellate cell, hepatocellular carcinoma, mitochondrial dynamics, mitochondrial transfer

## Abstract

The tumor margin of hepatocellular carcinoma (HCC) is a critical zone where cancer cells invade the surrounding stroma, exhibiting unique and more invasive metabolic and migratory features compared to the tumor center, driving tumor expansion beyond the primary lesion. Studies have shown that at this critical interface, HCC cells primarily rely on fatty acid oxidation to meet their energy demands, although the underlying mechanisms remain unclear. This study demonstrates that activated hepatic stellate cells (HSCs) at the tumor margin play a pivotal role in sustaining the metabolic needs of HCC cells. Specifically, it is discovered that mitochondrial fission regulator 2 (MTFR2) in HSCs interacts with dynamin‐related protein 1 (DRP1, a known mitochondrial fission machinery), preventing its lysosomal degradation, which in turn promotes mitochondrial fission. This MTFR2‐driven mitochondrial fission enhances the transfer of both fatty acids and mitochondria to HCC cells, supplying essential metabolic substrates and reinforcing the mitochondrial machinery critical for tumor growth. The findings suggest that targeting MTFR2‐driven mitochondrial fission may offer a novel therapeutic avenue for interfering with the metabolic crosstalk between tumor cells and the stromal niche.

## Graphical abstract

In the tumor margin of hepatocellular carcinoma (HCC), activated hepatic stellate cells upregulate MTFR2, initiating inhibiting DRP1 degradation and mitochondrial fission. This enhances mitochondrial availability and facilitates FAs synthesis via ACC1. Additionally, fission promotes RAC1‐mediated cytoskeletal remodeling and Miro1‐mediated mitochondrial transport, enabling HCC cells to utilize transferred resources for increased fatty acid oxidation and ATP production.

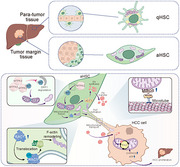

## 1 Introduction

Hepatocellular carcinoma (HCC) poses significant treatment challenges due to its complex tumor microenvironment (TME), characterized by metabolic heterogeneity and dynamic interactions at the invasive tumor margin.^[^ ^1^ ^]^ This critical interface, where HCC cells infiltrate adjacent tissues, exhibits a striking reliance on fatty acid oxidation (FAO) for energy production.^[^ ^2^ ^]^ However, the mechanisms driving this metabolic shift remain poorly elucidated. Understanding how HCC cells adapt to the unique microenvironmental conditions at the margin to favor FAO is pivotal for identifying exploitable therapeutic targets.

The metabolic reprogramming of cancer cells is intricately shaped by the TME's cellular composition, including immune and stromal cells, which engage in robust intercellular communication. This cross‐talk supplies growth factors, extracellular matrix components, nutrients, and signaling molecules that sustain tumor progression.^[^ ^1^, ^3^ ^]^ For example, macrophages can redirect cancer cell metabolism toward glycan pathways, enhancing their survival and proliferative capacity.^[^ ^4^ ^]^ Simultaneously, to fulfill their high energy demands and enhance metabolic flexibility, cancer cells exhibit a remarkable ability to hijack healthy mitochondria from neighboring tumor‐associated cells, which not only provides an immediate boost in ATP production but also improves mitochondrial quality, helping to counteract oxidative stress and resist cell death.^[^ ^5^ ^]^ Altogether, cells in the TME provide metabolic substrates and signaling support, thereby facilitating cancer metabolic reprogramming. In the context of the HCC margin, hepatic stellate cells (HSCs), a type of main lipid‐storing cell, are highly abundant and actively activated within the TME, supporting extracellular matrix deposition, immune evasion, and possibly providing metabolic substrates that promote a preference for FAO.^[^ ^6^ ^]^ Given that FAO depends on both fatty acids (FAs) as substrates and functional mitochondria as metabolic machinery, HCC cells may exploit activated HSCs (aHSCs) as a source of both. Investigating how HSC activation enhances FA availability and mitochondrial transfer to HCC cells is essential for unraveling the metabolic adaptations at the tumor margin.

Mitochondrial dynamics, defined by a delicate balance between fission and fusion, function not only as a “rocker switch” that modulates the transition from a quiescent to an activated state but also as an “effector” that drives changes during functional shifts in cellular behavior.^[^ ^7^ ^]^ Analysis of Gene Expression Omnibus (GEO) datasets in our study revealed that mitochondrial fission regulator 2 (MTFR2, gene name FAM54A) is uniquely and significantly upregulated during HSC activation among mitochondrial dynamics‐related genes. This suggests a central role for MTFR2 in aHSC‐mediated tumor progression at the HCC margin. Exploring how MTFR2 modulates mitochondrial dynamics and influences cancer cell metabolism is critical to understanding its contribution to tumor biology.

In this study, we demonstrate that MTFR2 is markedly elevated in aHSCs at the HCC margin, where it promotes mitochondrial fission and facilitates the transfer of FAs—key FAO substrates—and functional mitochondria to HCC cells. This dual provision of metabolic “fuel” and “factories” supports HCC cell proliferation and tumor expansion. Targeting MTFR2 to disrupt mitochondrial fission could impede the delivery of these vital metabolic resources, thereby curtailing tumor growth. This approach highlights a novel therapeutic strategy for HCC, leveraging metabolic vulnerabilities at the tumor‐stroma interface.

## 2 Results

### 2.1 MTFR2 Overexpression Drives Mitochondrial Fission in aHSCs and Supporting HCC Progression

**Figure 1:**
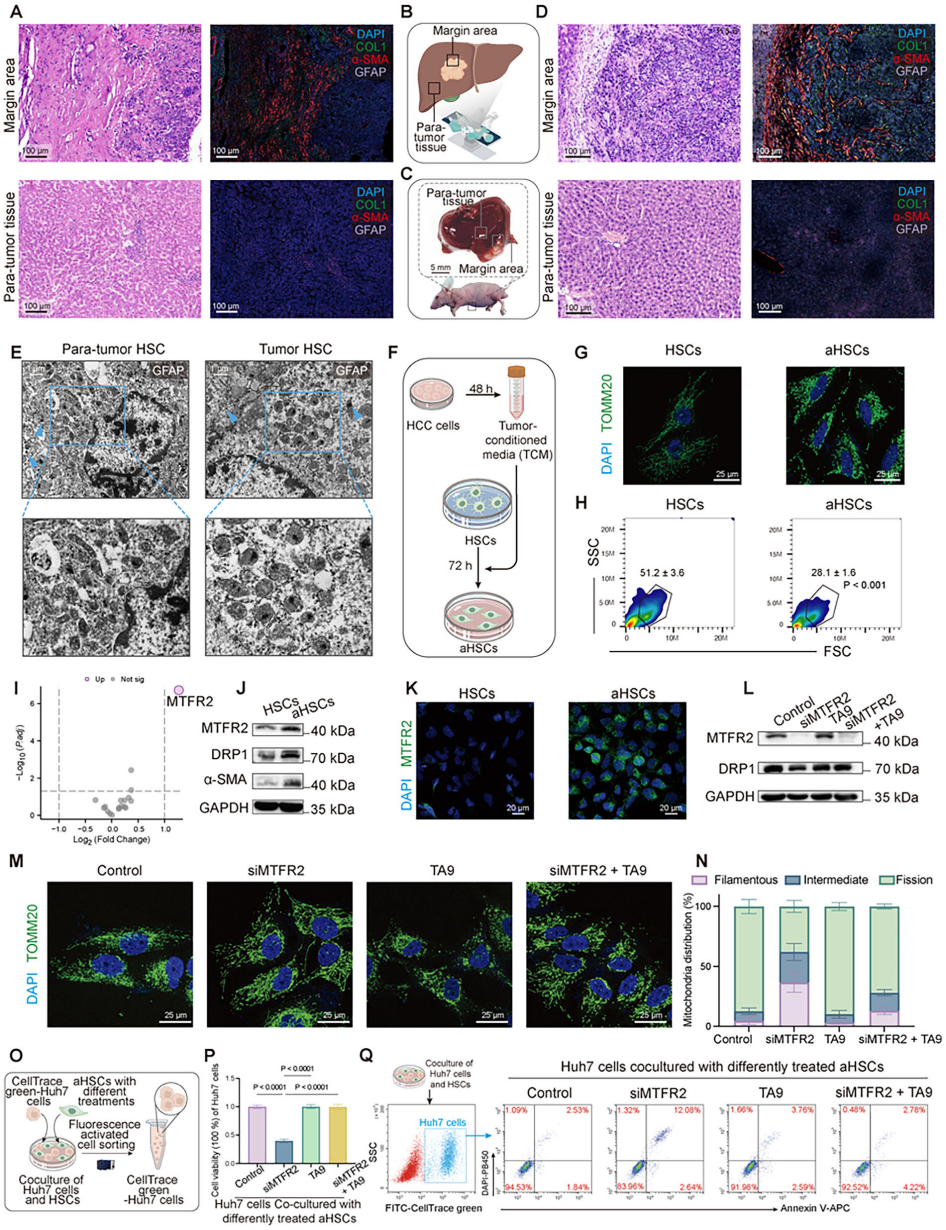
MTFR2‐mediated mitochondrial fission in aHSCs enhances HCC progression. A–D) Histological analysis of para‐tumor and tumor margin regions from HCC patient and mouse orthotopic HCC model. A) Hematoxylin and eosin (H&E) staining and immunofluorescence (IF) for COL1 (green), α‐SMA (red), and GFAP (purple, indicating HSC) in the margin area and para‐tumor tissue from an HCC patient. B) Schematic illustration indicating tissue collection sites for (A)—the margin area and para‐tumor tissue. C) Schematic for the tissue collection shown in (D), representing areas in a mouse orthotopic HCC model. D) H&E and IF staining of liver tissues to compare tumor margin and para‐tumor regions. Scale bars: 100 µm. E) Immunoelectron microscopy of GFAP indicating HSC from para‐tumor and tumor‐associated regions. Cyan arrow points to the gold particle bonded to GFAP. Scale bars: 1 µm. F) Diagram illustrating the experimental setup for activating HSCs. HSCs were treated with tumor‐conditioned media (TCM) for 72 h to induce activation. G,H) Confocal imaging and flow cytometry analysis of mitochondrial morphology of HSCs and aHSCs. G) IF staining of mitochondria using TOMM20 (green) in HSCs and aHSCs, indicating changes in mitochondrial distribution and morphology. Scale bars: 25 µm. H) Flow cytometry scatter plots showing side scatter (SSC) and forward scatter (FSC) for mitochondria of HSCs and aHSCs, highlighting mitochondrial size and granularity. The proportion of larger mitochondria (indicative of fused states) was quantified, with aHSCs showing a significant decrease compared to quiescent HSCs (*n* = 3). I) Volcano plot displaying the differential expression of mitochondrial dynamics‐related genes in quiescent HSCs and aHSCs. J) Western blot analysis of MTFR2, DRP1, and α‐SMA expression in HSCs and aHSCs. K) IF staining of MTFR2 (green) in HSCs and aHSCs, demonstrating increased MTFR2 expression in activated cells. Scale bars: 20 µm. L) Western blot results showing the effects of MTFR2 silencing (siMTFR2), the mitochondrial division inducer TA9, and the combination treatment on MTFR2 and DRP1 protein levels. GAPDH was used as the loading control. M,N) Confocal imaging and quantification of mitochondrial morphology under different conditions. Scale bars: 25 µm. Data are represented as mean ± SD (*n* = 10). O) Schematic of coculture experiments to assess the influence of aHSCs with different treatments to Huh7 cells. aHSCs treated with siMTFR2, TA9, or control conditions were cocultured with CellTrace green‐labeled Huh7 cells for 24 h, Huh7 was sorted out using flow cytometry. P) Cell viability of Huh7 sorted according to (O) (Data are represented as mean ± SD, *n* = 5, one‐way ANOVA was performed). Q) Flow cytometry apoptosis analysis of CellTrace green‐labeled Huh7 cells cocultured with differently treated aHSCs.

To explore the role of HSC activation within the HCC TME, we analyzed tissue samples from HCC patients and mouse models. Immunofluorescence (IF) staining (**Figure** **1**A–D and Figure S1, Supporting Information) revealed marked HSC activation at the tumor margin, characterized by elevated expression of collagen type I (COL1) and alpha‐smooth muscle actin (α‐SMA). In contrast, HSCs in para‐tumor regions remained largely quiescent with low expression of these markers. These findings, consistent with previous report,^[^ ^8^ ^]^ suggest that HSC activation occurs predominantly in the tumor margin. To assess pathological alterations in HSCs at tumor margins, we employed immunoelectron microscopy with glial fibrillary acidic protein (GFAP) as a specific HSC marker.^[^ ^9^ ^]^ This analysis demonstrated increased mitochondrial fission in HSCs from tumor regions compared to their quiescent counterparts in para‐tumor tissues (Figure 1E).

To confirm these mitochondrial morphological changes in vitro, we treated the human HSC line LX2 with tumor‐conditioned medium (TCM) to induce activation (Figure 1F), and the activation of HSCs was validated by upregulated α‐SMA expression (Figure S2A,B, Supporting Information). Fluorescence microscopy and flow cytometry analyses further revealed that aHSCs displayed small, punctate mitochondria (Figure 1G,H and Figure S2C, Supporting Information). Given these pronounced mitochondrial alterations, we analyzed transcriptomic datasets (GSE68001, GSE39469, GSE680000, GSE11954, GSE52234) from the GEO public database. Following batch effect correction (Figure S3A, Supporting Information), we screened mitochondrial dynamics‐related genes and identified mitochondrial fission regulator 2 (MTFR2) as the only gene significantly upregulated in aHSCs (Figure 1I). Western blot and IF analyses further confirmed elevated MTFR2 expression, alongside increased levels of the mitochondrial fission marker dynamin‐related protein 1 (DRP1), in aHSCs (Figure 1J,K and Figure S3B,C, Supporting Information). Similar results were observed in HSCs activated by TCM from MHCC‐97H cells (Figure S3D, Supporting Information).

To elucidate MTFR2's function, we used siRNA‐mediated silencing (efficacy validated in Figure S4A, Supporting Information) and tyrphostin A9 (TA9), a mitochondrial fission inducer (optimal concentration determined in Figure S4B–D, Supporting Information).^[^ ^10^ ^]^ Western blot analysis and confocal microscopy revealed MTFR2 knockdown in aHSCs reduced DRP1 expression and promoted elongated mitochondrial networks. Notably, TA9 treatment reversed these effects, restoring the fragmented mitochondrial morphology (Figure 1L–N and Figure S5A, Supporting Information). Given the importance of mitochondrial dynamics to mitochondrial metabolism,^[^ ^11^ ^]^ we further assessed the metabolic consequences of MTFR2 silencing through Seahorse analysis (Figure S5B–E, Supporting Information), which revealed a significant reduction in basal respiration, maximal respiration, and spare respiratory capacity in MTFR2‐depleted aHSCs. Importantly, TA9 treatment restored these mitochondrial bioenergetic functions, supporting MTFR2's role in mitochondrial dynamics and cellular metabolism. To evaluate the impact of these mitochondrial changes on HCC cells, we performed coculture experiments with Huh7 cells and differentially treated aHSCs (Figure 1O). MTFR2 knockdown in aHSCs significantly inhibited Huh7 cell proliferation and induced apoptosis, whereas TA9 treatment restored proliferation and inhibited apoptosis to levels comparable to the control group (Figure 1P,Q and Figure S5F, Supporting Information). Similar proliferative effects were observed in MHCC‐97H cells (Figure S5G, Supporting Information).

Collectively, these results demonstrated that MTFR2 upregulation in aHSCs promoted mitochondrial fission, thereby influencing the behavior of adjacent HCC cells. The capacity of MTFR2 to regulate aHSC mitochondrial dynamics and support HCC cell proliferation highlighted a pivotal interaction within the HCC TME.

### 2.2 MTFR2 of aHSCs Promotes HCC Progression by Orchestrating Mitochondrial Fission to Fuel Fatty Acid Oxidation in HCC Cells

**Figure 2:**
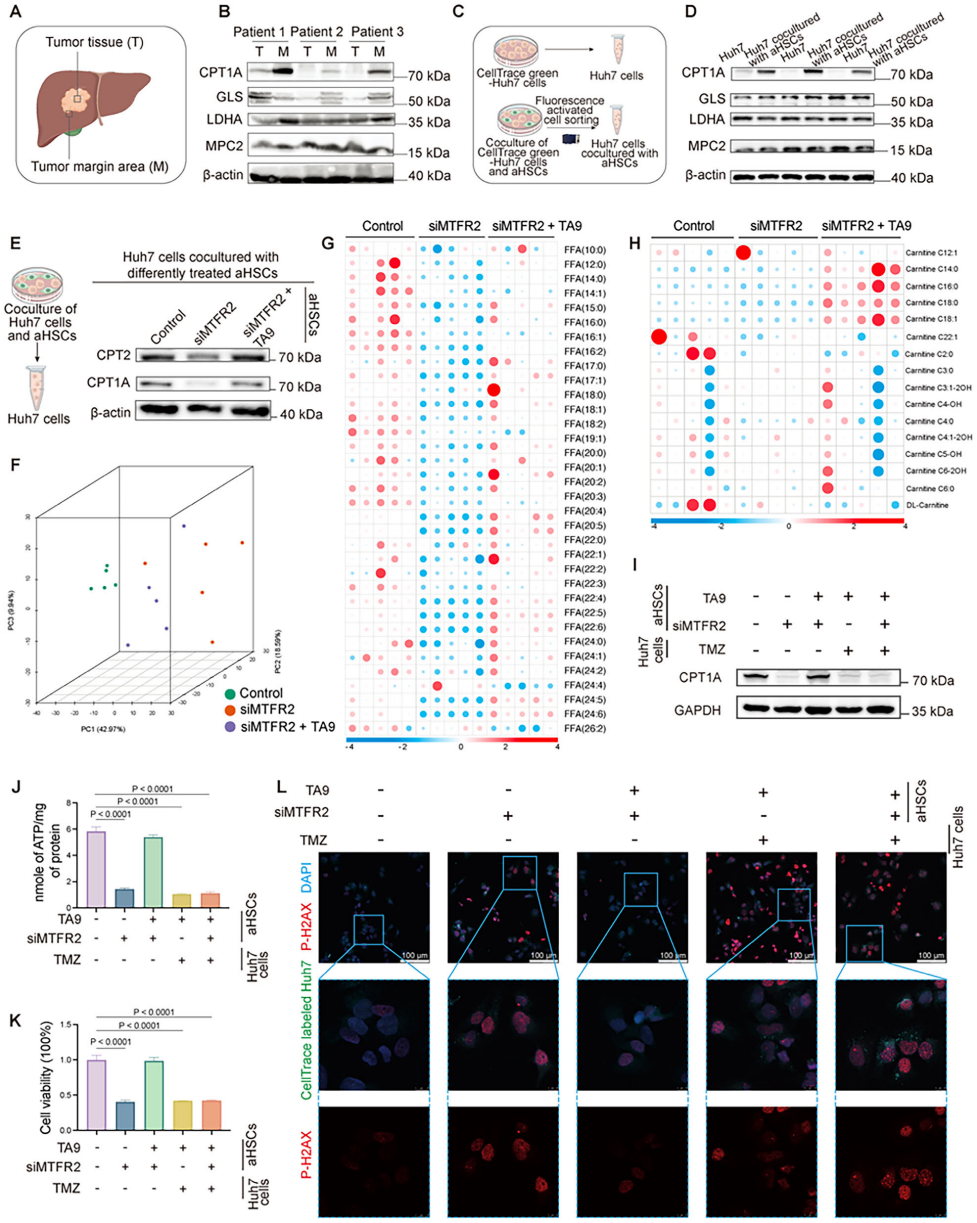
MTFR2‐mediated mitochondrial fission in aHSCs promotes fatty acid oxidation (FAO) in HCC cells and enhancing HCC progression. A) Schematic diagram of tumor tissue sampling. B) Western blot analysis of key mitochondrial metabolic enzymes in tumor (T) and tumor margin (M) areas as (A) suggesting from three HCC patients. C) Experimental design comparing Huh7 cells cultured alone versus cocultured with HSCs for 24 h. D) The protein level of key mitochondrial metabolic enzymes between Huh7 alone and Huh7 cocultured with aHSCs showing by western blot. E) CPT1A and CPT2 expression level in Huh7 cells cocultured with different treated aHSCs. F) Principal Component Analysis (PCA) plot showing metabolic profiles of three experimental groups. G) Heatmap shows differential FA contents. H) Heatmap shows relative difference in carnitine. I) Western blot analysis of CPT1A expression in Huh7 cells cocultured with aHSCs under different treatment conditions. J) Quantification of ATP production in Huh7 cells cocultured with aHSCs under different treatment conditions (Data are represented as mean ± SD, *n* = 3, one‐way ANOVA was performed). K) CCK‐8 cell proliferation assay in Huh7 cells cocultured with aHSCs under different treatment conditions (Data are represented as mean ± SD, *n* = 4, one‐way ANOVA was performed). L) Immunofluorescence staining of P‐H2AX (suggesting the damaged DNA, red). CellTrace green‐labeled Huh7 (green) cocultured with aHSCs under different treatment conditions. Scale bars: 100 µm.

Metabolic reprogramming is essential for tumor progression, particularly within the distinct TME shaped by various stromal components, where tumor cells adapt to meet their metabolic demands. Our study identified FAO as a predominant metabolic pathway at the HCC tumor margin. Notably, carnitine palmitoyl transferase 1A (CPT1A), a rate‐limiting enzyme in FAO, exhibited significant upregulation in this region (**Figure** **2**A,B and Figure S6A, Supporting Information). Since aHSCs are more abundant at the tumor margin, we investigated their role in driving this metabolic shift. Coculture experiments revealed that CPT1A expression was markedly elevated in Huh7 cells when cultured with aHSCs (Figure 2C,D and Figure S6B, Supporting Information), underscoring the metabolic influence tending to FAO exerted by aHSCs on tumor cells.

Building on our prior observation that MTFR2 is overexpressed in aHSCs and promotes tumor cell proliferation via mitochondrial fission, we hypothesized that this effect is mediated through enhanced FAO. To test this, we silenced MTFR2 in aHSCs using siRNA and restored mitochondrial fission with TA9 (Figure S7A, Supporting Information). These processed aHSCs were then cocultured with Huh7 or MHCC‐97H cells for 24 h (Figure S7B, Supporting Information). MTFR2 knockdown in aHSCs led to reduced expression of key FAO enzymes, CPT1A and carnitine palmitoyl transferase 2 (CPT2) in HCC cells, while TA9 treatment restored their levels (Figure 2E and Figure S7C,D, Supporting Information). Lipidomic analysis further confirmed decreased FAs and carnitine levels in Huh7 cells cocultured with MTFR2‐silenced aHSCs (Figure 2F–H), underscoring a direct link between aHSC MTFR2 expression and HCC cell FAO activity (Figure S7E, Supporting Information).

To evaluate whether the pro‐tumorigenic effect of aHSCs depend on FAO, we inhibited FAO in HCC cells treated trimetazidine dihydrochloride (TMZ, 10 × 10^−6^ m).^[^ ^12^ ^]^ Our results showed that even treating aHSCs with TA9, the restored expression of CPT1A, cellular viability, and ATP production in HCC cells were all reduced again when FAO was inhibited (Figure 2I–K and Figure S8A, Supporting Information). Comparable outcomes were observed in MHCC‐97H coculture systems (Figure S8C,D, Supporting Information). Given FAO's role in energy supply, oxidative stress management, and DNA repair,^[^ ^13^ ^]^ we assessed DNA damage via phosphorylated histone H2AX (P‐H2AX), a marker of double‐strand breaks.^[^ ^14^ ^]^ In Huh7 cells cocultured with MTFR2‐silenced aHSCs, P‐H2AX levels rose significantly, an effect reversed by TA9 treatment. However, FAO inhibition in Huh7 cells exacerbated DNA damage (Figure 2L and Figure S8E, Supporting Information).

These findings collectively demonstrate that MTFR2‐driven mitochondrial fission in aHSCs enhances FAO in HCC cells, fueling energy production and mitigating DNA damage, thereby promoting HCC progression. This interplay between aHSC mitochondrial dynamics and HCC metabolism unveils a critical mechanism at the tumor‐stroma interface.

### 2.3 Activation of HSCs Increases FAs Synthesis and Transfer to Cancer Cells, Driving Proliferation

**Figure 3:**
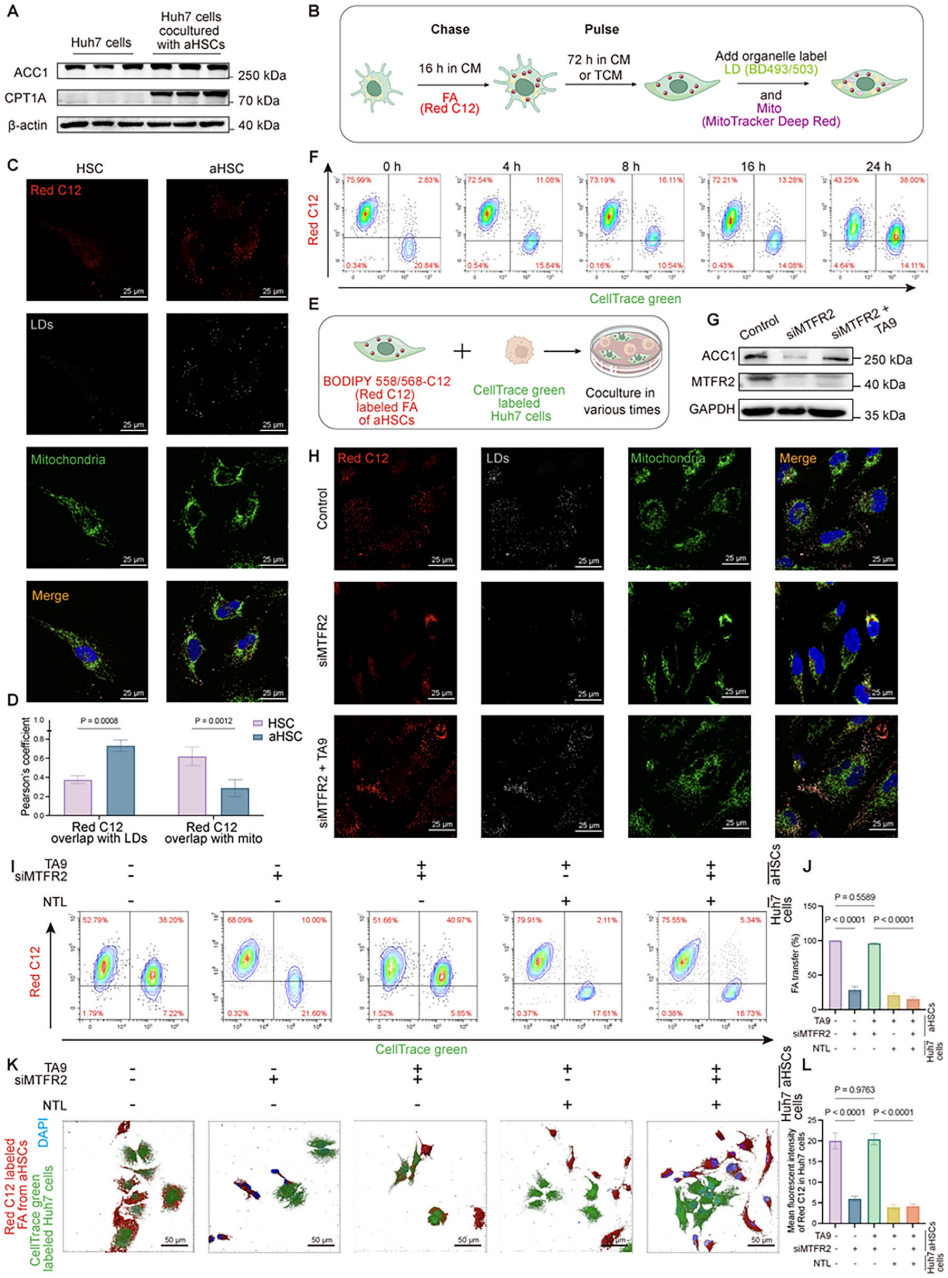
Activated HSCs enhance fatty acid (FA) synthesis and transfer to cancer cells, promoting proliferation. A) Western blot showing the ACC1 and CPT1A level of Huh7 cells and Huh7 cells cocultured with aHSCs (*n* = 3). B) Schematic of the experimental setup for FA chase pulse assay. HSCs were incubated in complete media (CM) with red fluorescent FA (Red C12) for 16 h (“pulse”). Following this, HSCs were cultured for an additional 72 h in CM or tumor‐conditioned media (TCM) without the labeled FA (“chase”). Mitochondria and lipid drops (LDs) were stained before imaging. C,D) FA localization was assayed as described in (A) and chased in CM or TCM. C) LDs were labeled using BODIPY 493/503 (gray) and mitochondria were labeled using MitoTracker Far Red (green). Scale bar = 25 µm. D) Relative cellular localization of Red C12 was quantified by Pearson's coefficient analysis (Data are represented as mean ± SD, *n* = 3, at least 50 cells analyzed per replicate, two‐way ANOVA was performed). E) Schematic representation of the coculture system used to track FA transfer from HSCs to Huh7 cells over various time points. BODIPY 558/568‐C12 labeled FA in HSCs and CellTrace green‐labeled Huh7 cells were used for tracking. F) Flow cytometry analysis showing the progressive transfer of FA from HSCs to Huh7 cells at different time points (0, 4, 8, 16, and 24 h). G) Western blot showing increased expression of ACC1 (Acetyl‐CoA carboxylase 1) in aHSCs, indicative of enhanced FA synthesis. H) Immunofluorescence (IF) images of FA pulse‐chase assay under different treatments. Scale bars: 25 µm. I,J) Flow cytometry analysis and its quantification further confirm the FA transfer to Huh7 cells when HSCs were treated with siMTFR2 or TA9 and Huh7 were treated with NTL (Data are represented as mean ± SD, *n* = 3, one‐way ANOVA was performed). K,L) IF 3D images reconstructed by Imaris and quantifications showing cocultured HSCs (Red C12‐labeled FA) and Huh7 cells (CellTrace green‐labeled) with differential treatment (Data are represented as mean ± SD, *n* = 6, at least 50 recipient cells analyzed per replicate, one‐way ANOVA was performed). Scale bar: 50 µm.

Sufficient FAs are essential for fueling FAO, prompting us to investigate their sources. Cancer cells can synthesize FAs de novo or acquire them from the surrounding TME.^[^ ^15^ ^]^ As the expression of acetyl‐CoA carboxylase 1 (ACC1), an essential enzyme in FAs synthesis, showed no difference between Huh7 cells cultured alone and with HSCs, while FAO key enzyme—CPT1A—significantly upregulated after coculturation (**Figure** **3**A and Figure S9A, Supporting Information). This suggests that Huh7 cells predominantly rely on exogenous FA sources, likely from aHSCs in the TME.

HSCs, primarily situated in the liver's perisinusoidal space and recognized for their role in fibrosis,^[^ ^16^ ^]^ undergo activation in HCC, particularly at tumor margins. These aHSCs secrete growth factors, cytokines, and extracellular matrix components that bolster tumor growth, angiogenesis, and metastasis.^[^ ^17^ ^]^ Our study revealed that TCM triggers ACC1 upregulation in aHSCs, resulting in elevated FA production (Figure S9B, Supporting Information). Considering the utilization of FAs in mitochondria or their storage in lipid droplets (LDs), a balance is achieved in cellular energy management,^[^ ^18^ ^]^ we utilized a pulse‐chase assay to track FAs in relation to LDs, the FAs conduit, and mitochondria, the FAs factory, in HSCs (Figure 3B).^[^ ^19^ ^]^ BODIPY 558/568 C12 (Red C12), a saturated FAs analog with a 12‐carbon tail, was used to track the FAs.^[^ ^20^ ^]^ HSCs were labeled overnight with Red C12 (1 × 10^−3^ m), followed by incubation in normal medium or TCM for 72 h. Confocal microscopy, with LDs stained by BODIPY 493/503 and mitochondria by MitoTracker Far Red, showed that quiescent HSCs in (complete medium, CM) retained Red C12 predominantly in mitochondria. In contrast, aHSCs in TCM exhibited a marked shift of Red C12 from mitochondria to LDs (Figure 3C,D), indicating storage of excess FAs in LDs, potentially for subsequent release within the TME.

Given HCC cells’ dependence on external FAs and aHSCs’ proximity and FA reserves, we tested whether aHSCs transfer FAs to support cancer cell proliferation. Using a coculture system, aHSCs labeled with Red C12 were paired with Huh7 cells marked with a green tracker (Figure 3E). Flow cytometry confirmed significant Red C12 transfer to Huh7 cells over time (Figure 3F), supporting the notion that aHSCs supply FAs to fuel HCC growth.

To evaluate the correlation between MTFR2 and FAs synthesis, localization and expulsion, we performed the same experiments in aHSCs with MTFR2 knockdown and TA9 treatment to restore mitochondrial morphology. As shown in Figure 3G,H and Figure S10 (Supporting Information), limiting MTFR2 expression resulted in the downregulation of ACC1, reduction in FAs levels, decreased FAs accumulation in LDs and declined FA transfer to Huh7 cells. These effects were reversed by TA9 treatment, which promoted mitochondrial fission.

To confirm the dependence of HCC progression on MTFR2‐mediated FA transfer, we inhibited FA uptake in Huh7 and MHCC‐97H cells with nortriptylin hydrochloride (NTL),^[^ ^21^ ^]^ with efficacy validated (Figure 3I–L and Figure S11, Supporting Information). Despite TA9 restoring FA transfer from aHSCs, NTL treatment diminished Huh7 proliferation (cell counting kit‐8 [CCK‐8] assay) and increased MHCC‐97H apoptosis, indicating FA transfer's critical role in tumor growth (Figure S12A–C, Supporting Information). However, NTL‐treated Huh7 cells still exhibited higher proliferation and ATP production compared to those cultured alone (Figure S12D,E, Supporting Information), suggesting aHSCs contribute additional growth‐supporting factors beyond FAs.

### 2.4 MTFR2‐Mediated Mitochondrial Fission in HSCs Facilitates Mitochondrial Transfer to HCC Cells, Enhancing Tumor Proliferation

**Figure 4:**
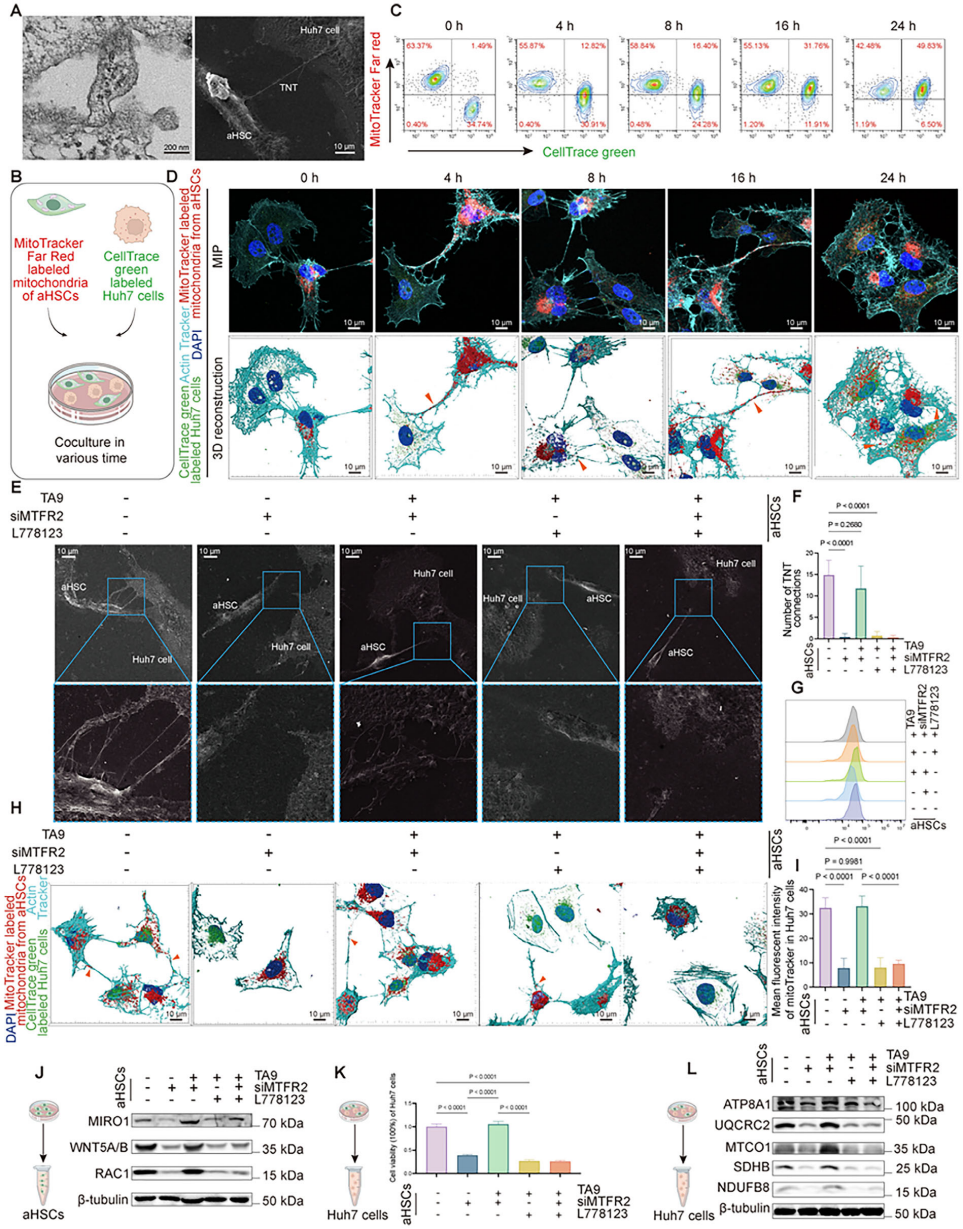
MTFR2‐driven mitochondrial fission in aHSCs facilitates mitochondrial transfer to Huh7 cells, boosting tumor progression. A) Transmission electron microscopy (TEM) (left panel, scale bar: 200 nm) and scanning electron microscopy (SEM) (right panel, scale bar: 10 µm) images showing direct contact between aHSCs and Huh7 cells. B–D) Mitochondrial transfer from aHSCs to Huh7 cells in various coculture times. Illustrate representation of the coculture system used to track mitochondrial transfer from HSCs (MitoTracker Far Red‐labeled) to Huh7 cells (CellTrace green‐labeled) at various time points (B). Flow cytometry of mitochondrial transfer efficiency (C). Spatiotemporal visualization of mitochondrial transfer (D). Upper panels: Maximum intensity projections (MIPs) of confocal Z‐stacks showing time‐dependent accumulation of aHSC‐derived mitochondria (red) in Huh7 cells (green). Lower panels: 3D surface rendering (Imaris) of recipient Huh7 cells. Orange arrow points to the mitochondria from aHSCs in TNTs toward to Huh7 cells. Scale bars: 10 µm. E–L) Coculture of Huh7 cells with aHSCs with different treatments. E) Scanning electron microscopy (SEM) images showing nanotubes between Huh7 cells and aHSCs (scale bar: 10 µm). F) Graphs showing the numbers of nanotubes connecting the Huh7 and HSCs, as calculated from the SEM images (Data are represented as mean ± SD, *n* = 7, one‐way ANOVA was performed). G) Histograms of fluorescence in Huh7 cells (CellTrace green‐labeled) showing the uptake of mitochondria from cocultured aHSCs. H) Representative 3D confocal image showing nanotube formation and MitoTracker‐labeled mitochondrial transfer (orange arrows) from aHSCs and the quantification (I) (Data are represented as mean ± SD, *n* = 6, at least 50 recipient cells analyzed per replicate, one‐way ANOVA was performed). Scale bar: 10 µm. J) Western blot analyzing the protein expression levels of MIRO1, WNT5A/B, and RAC1 in aHSCs with different treatments. K) Cell viability of Huh7 cells sorted by flow cytometry (Data are represented as mean ± SD, *n* = 4, one‐way ANOVA was performed). L) Analysis of the main protein levels of mitochondrial respiratory chain in Huh7 coculturing with various treatments of aHSCs.

In order to investigate additional support provided by aHSCs to Huh7 cells, we used transmission electron microscopy (TEM) and scanning electron microscopy (SEM) to explore the intercellular communication, which revealed tunneling nanotubes (TNTs) connecting aHSCs and Huh7 cells (**Figure** **4**A). Given that TNTs are established conduits for mitochondrial transfer from stromal to cancer cells, boosting tumor bioenergetic capacity,^[^ ^22^ ^]^ we hypothesized that aHSCs deliver mitochondria to HCC cells via this route. To test this, we labeled mitochondria within aHSCs with MitoTracker Far Red and cocultured them with CellTrace green‐labeled HCC cells over different time intervals (Figure 4B). Flow cytometry and confocal microscopy collectively confirmed a time‐dependent accumulation of aHSC‐derived mitochondria in Huh7 cells (Figure 4C,D and Figure S13, Supporting Information).

To ascertain whether MTFR2‐driven HCC progression relies on mitochondrial transfer, we first identified the transfer pattern. Given prior evidence that mitochondrial transfer primarily occurs through TNTs or extracellular vesicles,^[^ ^23^ ^]^ we established a compartmentalized coculture model Huh7 cells (upper chamber) and aHSCs (lower chamber) were physically separated by 3 µm pores, allowing vesicular communication but blocking TNT formation (Figure S14, Supporting Information). This setup reduced mitochondrial transfer compared to direct coculture, establishing TNTs as the predominant pathway. We then optimized the TNT inhibitor L778123 (inhibitor of TNT forming^[^ ^24^ ^]^) concentration, finding that 10 × 10^−6^ m effectively blocked mitochondrial trafficking without compromising aHSC viability (Figure S15, Supporting Information).

Next, we explored MTFR2's regulatory role in mitochondrial transfer. SEM, confocal microscopy, and flow cytometry demonstrated that MTFR2 knockdown in aHSCs diminished TNT formation, an effect reversed by TA9 treatment. However, L778123 abrogated mitochondrial transfer even with TA9 present (Figure 4E–I and Figure S16A, Supporting Information), with consistent results in MHCC‐97H cocultures (Figure S16B,C, Supporting Information). Mechanistically, MTFR2 modulated the expression of TNT‐associated proteins: Wnt family member 5A/B (WNT5A/B), which initiates cytoskeletal rearrangement for TNT formation;^[^ ^25^ ^]^ RAC1, which stabilizes TNTs; and MIRO1, which facilitates mitochondrial movement along TNTs.^[^ ^26^ ^]^ These proteins’ expression levels correlated with MTFR2 (Figure 4J and Figure S17A, Supporting Information), suggesting MTFR2 acts upstream to coordinate TNT‐mediated mitochondrial transfer.

To evaluate the impact on HCC progression, we cocultured aHSCs and HCC cells under varying conditions, sorting HCC cells post‐coculture via flow cytometry. CCK‐8 assays showed that inhibiting mitochondrial transfer with L778123 significantly reduced HCC cell viability, an effect TA9 could not reverse (Figure 4K and Figure S17B, Supporting Information). Similarly, expression of key mitochondrial proteins in Huh7 cells mirrored these changes (Figure 4L and Figure S17C, Supporting Information), highlighting MTFR2‐driven mitochondrial transfer as a critical supporter of tumor metabolism and proliferation.

### 2.5 Synergistic Roles of FAs and Mitochondrial Transfer in Supporting HCC Cell Survival

**Figure 5:**
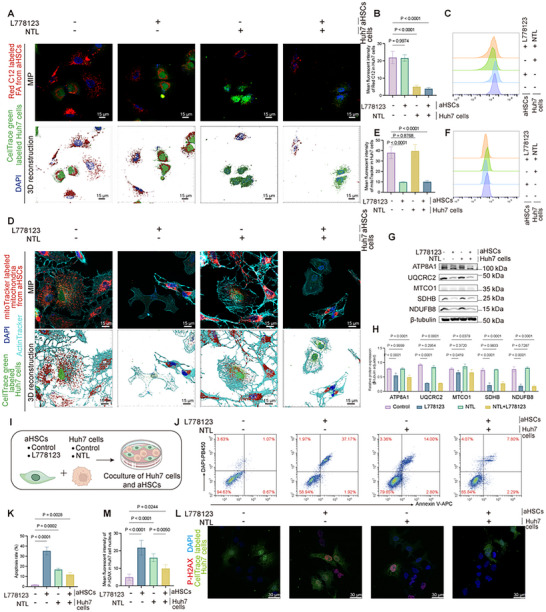
Complementary roles of FAs transfer and mitochondrial transport in supporting tumor cell survival. A) Representative confocal images of Huh7 cells cocultured with aHSCs under L‐778123 and NTL treatment. Maximum intensity projections (MIP) of confocal Z‐stacks showing Huh7 cells (CellTrace green, green) cocultured with aHSCs pre‐labeled with Red C12 fluorescent FA (red) (Upper panels). Corresponding 3D surface renderings (Imaris) of the boxed regions (Lower panels). Scale bars: 15 µm. B) Graph showing the fluorescence intensity of FAs in CellTrace green labeled Huh7 cells (Data are represented as mean ± SD, *n* = 6, at least 50 recipient cells analyzed per replicate, one‐way ANOVA was performed). C) Flow cytometry analysis of fluorescence intensity of FAs in FITC‐positive Huh7 cells. D) Confocal images showing the transportation of mitochondria from aHSCs to Huh7 cells. (Upper panels) MIP: Confocal Z‐stacks of cocultured MitoTracker‐labeled mitochondria in aHSCs (red) and CellTrace green‐labeled Huh7 cells (green), with ActinTracker (cyan) highlighting tunneling nanotubes (TNTs). (Lower panels) 3D surface reconstruction (Imaris). Scale bars: 15 µm. E) Quantification of fluorescence intensity from Figure D, showing the uptake of HSC‐derived mitochondria by Huh7 cells (Data are represented as mean ± SD, *n* = 6, at least 50 recipient cells analyzed per replicate, one‐way ANOVA was performed). F) Fluorescence histograms of Huh7 cells (labeled with CellTrace green) illustrating the mitochondria uptake from cocultured HSCs. G,H) Western blot analysis showing the expression of mitochondrial respiratory chain proteins in Huh7 cells following coculture with aHSCs and quantification (H) (Data are represented as mean ± SD, *n* = 3, two‐way ANOVA was performed). I) Schematic illustration of the experimental setup for coculturing aHSCs and Huh7 cells under different conditions, including L‐778123 to inhibit TNT formation and NTL to block Huh7 cell FA uptake. J,K) Flow cytometry analysis of apoptosis in Huh7 cells after coculture with HSCs under different treatments. Representative dual staining density plots (J) and quantification of total apoptotic cells (K) (Data are represented as mean ± SD, *n* = 3, one‐way ANOVA was performed). L,M) DNA damage assessment in Huh7 cells under differential treatments. Representative immunofluorescence images of P‐H2AX foci (red) in CellTrace green‐labeled Huh7 cells (green) with DAPI nuclear counterstain (blue) (L). Quantification of P‐H2AX mean fluorescence intensity in Huh7 cell nucleus (M) (Data are represented as mean ± SD, *n* = 3, at least 50 Huh7 cells analyzed per replicate, one‐way ANOVA was performed).

Further investigation was carried out to explore whether MTFR2 simultaneously triggers FAs transfer and mitochondrial transport to HCC cells, and whether these processes work synergistically. As illustrated in **Figure** **5**A–H and Figure S18 (Supporting Information), mitochondrial transport from aHSCs was reduced by L778123 and FAs support was blocked by treating HCC cells with NTL as indicated in Figure 5I. Individually inhibiting mitochondrial transfer (via L778123) or FA supply (via NTL) led to a significant increase in apoptosis and DNA damage while reducing tumor spheroid proliferation. However, under dual inhibition, the inhibitory effects were attenuated (Figure 5J–L and Figure S19A–D, Supporting Information), indicating that blocking one pathway alone imposes greater metabolic stress than blocking both. To elucidate this interplay, dihydroethidium (DHE) staining revealed significantly elevated ROS production in HCC cells upon mitochondrial transfer inhibition alone (Figure S19E,F, Supporting Information), suggesting that mitochondria from aHSCs alleviate oxidative stress by detoxifying FA‐derived lipid peroxides through enhanced β‐oxidation. The reduced ROS and cytotoxicity under dual inhibition highlight a compensatory relationship, where the absence of one process exacerbates the loss of the other. These results collectively demonstrate the complementary supportive roles of mitochondrial and FA transfer in promoting HCC cell survival.^[^ ^27^ ^]^

Our findings reveal that MTFR2‐coordinated FA and mitochondrial transfer function as interdependent metabolic modules that sustain HCC survival, with their synergistic interaction mitigating vulnerabilities associated with single‐pathway disruption.

### 2.6 MTFR2 in HSCs Enhances Tumor Growth In Vivo

**Figure 6:**
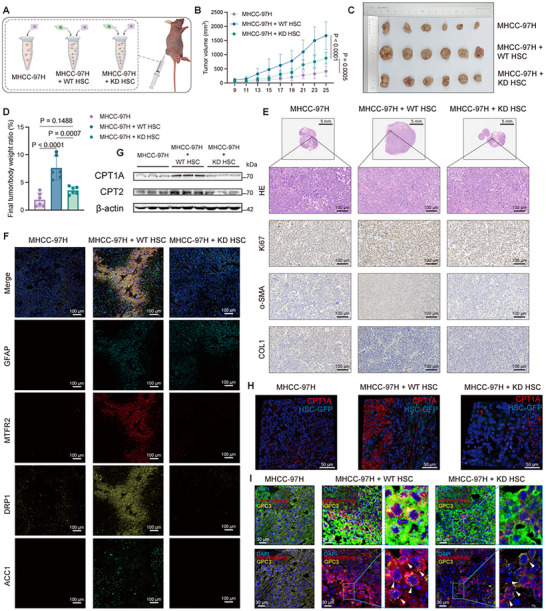
MTFR2 in HSCs drives tumor progression in vivo. A) Schematic illustration showing the experimental setup for tumor induction with MHCC‐97H cells and different HSC conditions. B) Graph depicting tumor volume over time for MHCC‐97H, MHCC‐97H + WT HSC, and MHCC‐97H + KD HSC groups (Data are represented as mean ± SD, *n* = 6, two‐way ANOVA was performed). C) Images of tumor nodules formed from MHCC‐97H cells co‐cultured with either wild‐type (WT) HSCs or knockdown (KD) HSCs. D) Analysis of the final tumor body weight ratio (%) (Data are represented as mean ± SD, *n* = 6, one‐way ANOVA was performed). E) Histological assessment via hematoxylin and eosin (H&E) staining, and immunohistochemical analysis of Ki67, α‐SMA, and COL1 in tumor sections. Scale bars: 100 µm. F) Immunofluorescence staining of various proteins (GFAP, MTFR2, DRP1, ACC1) in tumor sections, indicating expression patterns. Scale bars: 100 µm. G) Western blot analysis of CPT1A and CPT2 expression. H) IF staining of CPT1A in tumor tissues of different groups. GFP‐HSC was shown to distinguish HSCs. Scale bars: 50 µm. I) Fluorescent images showing the mito‐mcherry labeled mitochondria from HSCs in GPC3 positive MHCC‐97H cells (white arrows). Scale bars: 30 µm.

To investigate the role of HSC‐derived MTFR2 in HCC growth, MTFR2 knockdown (KD) HSCs were constructed by shRNA, and the optimal sequence was selected via Western blot (Figure S20, Supporting Information). MHCC‐97H cells were co‐injected subcutaneously with either wild‐type (WT) or KD HSCs into mice (**Figure** **6**A). Importantly, no significant differences in body weight were observed among the experimental groups (Figure S21A, Supporting Information). However, tumor weight and volume were significantly greater in mice co‐injected with MHCC‐97H cells and WT HSCs compared to those with MHCC‐97H cells alone, while MTFR2 depletion impaired the ability of HSCs to enhance tumor growth (Figure 6B–D). Additionally, tumor tissues from mice injected with HCC alone or co‐injected with MTFR2‐deficient HSCs displayed fewer Ki67‐positive cells compared to those co‐injected with WT HSCs (Figure 6E and Figure S21B, Supporting Information). Moreover, subcutaneous tumors from MHCC‐97H cells co‐injected with MTFR2‐deficient HSCs exhibited lower levels of α‐SMA and COL1 compared to tumors co‐injected with WT HSCs, suggesting that MTFR2 deficiency in HSCs diminishes their capacity to promote tumor growth in vivo.

Multiplex immunofluorescence confirmed that the expression of MTFR2, DRP1, and ACC1 from HSCs was significantly impaired following MTFR2 knockdown (Figure 6F and Figure S21C, Supporting Information). Tumor tissue from the margin area was further analyzed to assess the impact of MTFR2 on FAO. The results revealed that key FAO enzymes, CPT1A and CPT2, were elevated following co‐injection, while MTFR2 depletion resulted in a reduction in these enzyme levels (Figure 6G,H and Figure S21D–F, Supporting Information). Fluorescence imaging also demonstrated a marked decrease in mitochondrial transfer when MTFR2 was suppressed (Figure 6I and Figure S21G, Supporting Information). Taken together, these findings establish MTFR2 in HSCs as a critical driver of HCC progression, promoting mitochondrial fission and accelerating FAO in HCC cells.

### 2.7 MTFR2 Promotes HCC Progression via DRP1 Stabilization and Coordinated Mitochondrial and FAs Transfer

**Figure 7:**
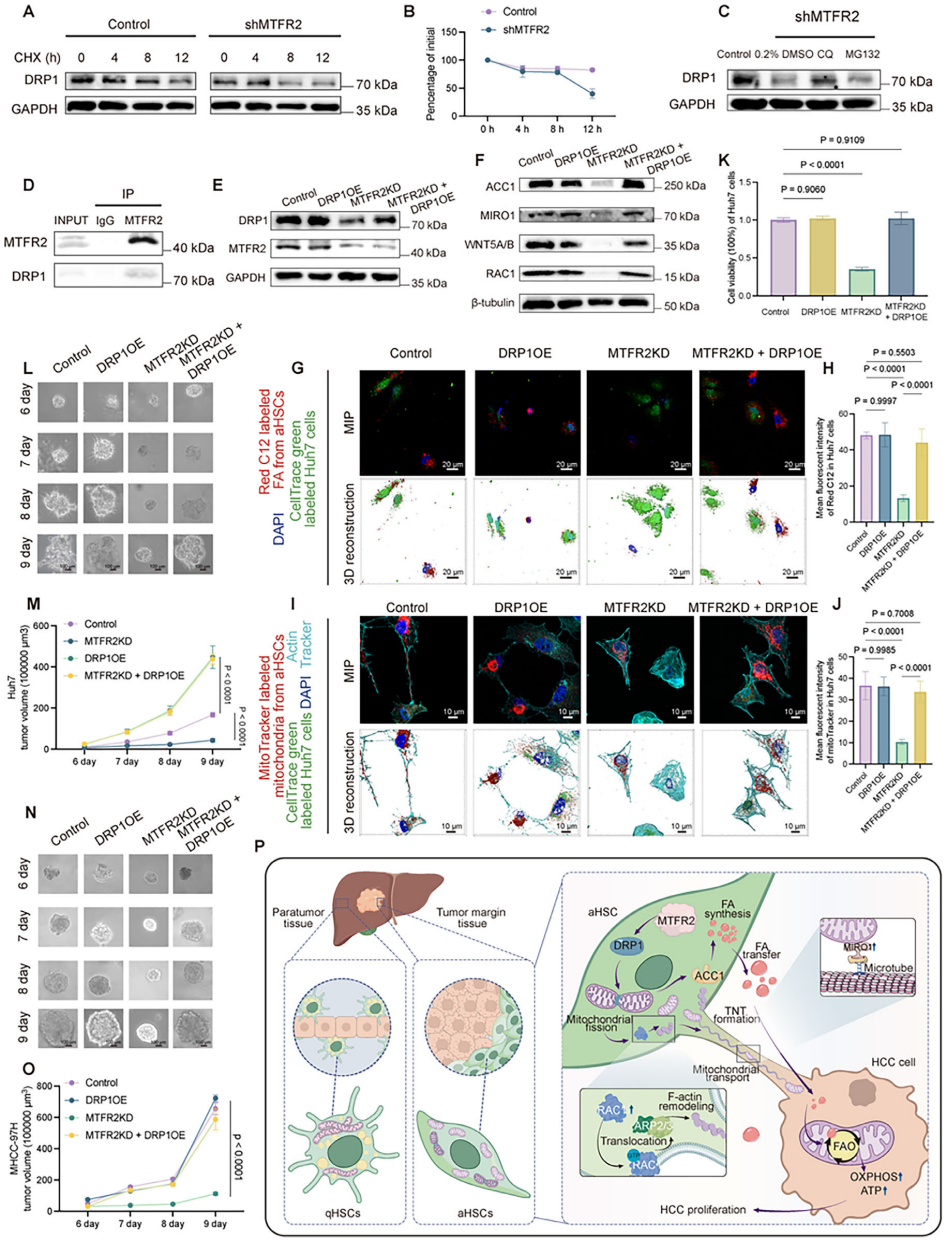
MTFR2‐dependent proliferation via DRP1. A,B) Cycloheximide (CHX) chase assay (*n* = 3), DRP1 levels were normalized to initial and shown in the graph (B). C) The expression level of DRP1 after different treatment: chloroquine (CQ, 50 × 10^−6^ m, 10 h) and MG132 (10 × 10^−6^ m, 10 h). D) Co‐immunoprecipitation (Co‐IP) assay to detect the interaction between MTFR2 and DRP1. E) Western blot analysis of the expression of MTFR2 and DRP1. F) Western blot analysis. G,H) Confocal images (upper panels: MIP; lower panels: 3D reconstruction, scale bar: 20 µm) showing the transportation of FAs from aHSCs with MTFR2 knocking down, DRP1 overexpression to Huh7 cells and quantification of the FA intensity in Huh7 cells (Data are represented as mean ± SD, *n* = 6, at least 50 recipient cells analyzed per replicate, one‐way ANOVA was performed). I,J) Mitochondrial transfer from aHSCs with different genetic manipulations to Huh7 cells. I) MIP images (upper panels) of confocal Z‐stacks and 3D surface rendering (Imaris) iamges (scale bar: 10 µm) showing aHSC‐derived mitochondria (mitoTracker, red) transferred to Huh7 cells (cellTrace green, green). J) Quantification of mean fluorescent intensity of mitoTracker in Huh7 cells (Data are represented as mean ± SD, *n* = 6, at least 50 recipient cells analyzed per replicate, one‐way ANOVA was performed). K) Cell viability of Huh7 cells cocultured with aHSCs under various conditions (Data are represented as mean ± SD, *n* = 4, one‐way ANOVA was performed). L,M) Representative images of tumor spheroid of Huh7 cells combined with aHSCs over times (Data are represented as mean ± SD, *n* = 6, two‐way ANOVA was performed) and calculation of the spheroid volume over times (M, Data are represented as mean ± SD, *n* = 6, one‐way ANOVA was performed). N,O) Dynamics of 3D tumor spheroid growth in MHCC‐97H/aHSC cocultures. N) Representative bright‐field images of spheroids in 6–9 d culture period (scale bars: 100 µm). O) Quantification of spheroid volume progression (Data are represented as mean ± SD, *n* = 6, two‐way ANOVA was performed) and calculation of the spheroid volume over times (O, Data are represented as mean ± SD, *n* = 6, one‐way ANOVA was performed). P) Simplified model depicting the role of MTFR2 in regulation of HCC progression. Within the tumor microenvironment, aHSCs exploit MTFR2 to coordinate a dual metabolic cascade that fuels HCC progression. MTFR2 upregulation in aHSCs triggers DRP1‐dependent mitochondrial fission. Subsequently, upregulating ACC1 facilitates FA synthesis and restoring FAs into LDs for transferring to HCC cells. At the same time, mitochondrial fission accelerates RAC1‐mediated remodeling of the actin cytoskeleton supports the structure and function of TNTs, and MIRO1 facilitating transporting mitochondria toward the forming TNTs. Consequently, HCC cells utilize the transferred FAs and mitochondria to enhance FAO, leading to increased ATP production.

Considering that DRP1 protein levels change following MTFR2 regulation and that DRP1 was crucial for mitochondrial fission, we assessed *DRP1* mRNA levels to further investigate the mechanism by which MTFR2 promotes mitochondrial fission. The results showed that *DRP1* mRNA levels remained unaffected (Figure S22, Supporting Information), suggesting that MTFR2 may regulate DRP1 at a post‐transcriptional level. MTFR2 knockdown accelerated the degradation of endogenous and exogenous DRP1 following cycloheximide (CHX) treatment, indicating that MTFR2 could extend the half‐life of DRP1 protein (**Figure** **7**A,B). To determine if MTFR2 inhibits DRP1 degradation through the ubiquitin‐proteasome or lysosomal pathway, aHSCs cells were treated with MG132 (a proteasome inhibitor) or chloroquine (CQ, a lysosome inhibitor) after MTFR2 downregulation. The shMTFR2‐mediated DRP1 destabilization was reversed by CQ but not MG132, indicating that MTFR2 stabilizes DRP1 via the lysosomal pathway (Figure 7C and Figure S23A, Supporting Information). At the same, Co‐IP assay demonstrated the interaction between MTFR2 and DRP1 (Figure 7D). To further elucidate the MTFR2‐DRP1 interaction, we used the AlphaFold3 server to simulate their binding mode, as shown in Figure S23B (Supporting Information), predicting the formation of 10 hydrogen bonds, which suggests a stable interaction that likely contributes to their functional relationship. Taken together, MTFR2 might combine with DRP1 and inhibit the lysosome‐dependent degradation, thereby promoting mitochondrial fission.

In investigating whether MTFR2‐mediated tumor progression depends on DRP1, we performed sequential genetic manipulations: first suppressing MTFR2 via shRNA‐mediated knockdown, then restoring DRP1 expression through plasmid overexpression (Figure 7E and Figure S24A, Supporting Information). As shown in Figure 7F and Figure S24B (Supporting Information), the expression of ACC1 (involved in fatty acid synthesis), mitochondrial transport‐related proteins such as RAC1, WNT5A/B, MIRO1, and DRP1 was synchronized. Confocal microscopy showed that inhibition of MTFR2 blocked both FAs and mitochondrial transfer from aHSCs to HCC cells. However, DRP1 overexpression restored these transfers (Figure 7G–J). Sorted cocultured Huh7 cells revealed that reduced MTFR2 expression in HSCs led to a significant decline in tumor cell proliferation, which was rescued by DRP1 overexpression (Figure 7K). Similarly, spheroid size analysis showed that DRP1 overexpression partially restored tumor growth that was impaired by MTFR2 knockdown (Figure 7L–O). These findings suggest that MTFR2 promotes tumor progression through a mechanism critically dependent on DRP1, highlighting the importance of mitochondrial dynamics in supporting tumor growth.

## 3 Discussion

This study investigates the interactions between HSCs and HCC cells at the tumor margin, a critical region where HCC progression is influenced by the TME. Specifically, we found that aHSCs surrounding HCC cells express elevated levels of MTFR2, which triggers mitochondrial fission. This process facilitates the transfer of FAs and mitochondria to HCC cells, enabling them to meet the metabolic demands of rapid proliferation through FAO. These findings underscore MTFR2‐driven mitochondrial dynamics as a potential therapeutic target to impede HCC development.

MTFR2, a member of the MTFR1 family of mitochondrial proteins alongside MTFR1 and MTFR1L, regulates mitochondrial fission during mitosis through mechanisms that remain incompletely understood.^[^ ^28^ ^]^ Our research demonstrates that MTFR2 promotes mitochondrial fission in aHSCs by stabilizing DRP1 via inhibition of its autophagic degradation. This aligns with prior studies showing MTFR2's role in tumor progression, such as in breast cancer and oral squamous cell carcinoma, where it shifts glucose metabolism toward glycolysis to enhance proliferation.^[^ ^29^ ^]^ In the context of HCC, we focused on MTFR2's function within the stromal TME, particularly in aHSCs. Upregulation of MTFR2 in aHSCs induces mitochondrial fission, whereas its knockdown significantly suppresses this process. Co‐culture experiments further revealed that disrupting MTFR2 in HSCs reduces HCC cell proliferation, suggesting that MTFR2 in aHSCs is integral to HCC progression. Given that HSCs are lipid‐rich and fragmented mitochondria are smaller and more readily transferable,^[^ ^30^ ^]^ we propose that MTFR2‐mediated mitochondrial fission enables aHSCs to supply both FAs and functional mitochondria to adjacent HCC cells, fostering their interaction and supporting tumor growth.

FAs are essential for HCC cell survival and proliferation, particularly at the tumor margin, where cells rely heavily on exogenous FAs to fuel energy production via β‐oxidation and synthesize membrane lipids for growth and division. This dependency highlights the critical interplay between HCC cells and aHSCs, which serve as a key source of metabolic substrates. Our data indicate that mitochondrial fission, driven by MTFR2, enhances FA transfer from aHSCs to HCC cells. Previous research has shown that fused mitochondria distribute FAs evenly across the mitochondrial network, optimizing β‐oxidation, whereas fragmented mitochondria lead to FA accumulation in LDs, reducing oxidative metabolism.^[^ ^19^ ^]^ In our study, this FA accumulation in LDs not only acts as an energy reservoir but also facilitates efficient transfer to HCC cells. Thus, mitochondrial fission not only maintains mitochondrial network integrity but also directly influences the efficiency of FA delivery from aHSCs, supporting HCC cell metabolism.

Beyond FA transfer, aHSCs contribute mitochondria to HCC cells, aiding in the management of exogenous FA influx, enhancing FAO, and mitigating lipotoxicity risks from FA buildup. This mitochondrial transfer is also initiated by MTFR2‐induced fission. Following fission, RAC1 activation stimulates WASP‐interacting protein (WIP), while elevated cytoplasmic Ca^2^⁺ levels inhibit WASP‐WIP droplet formation, exposing WIP phosphorylation sites and promoting cytoskeletal reorganization essential for TNT formation.^[^ ^31^ ^]^ TNTs establish physical connections between cells, and MIRO1 facilitates mitochondrial transport along microtubules toward these structures.^[^ ^32^ ^]^ The presence of WNT5A/B further amplifies this process by modulating pathways that regulate cell motility and cytoskeletal dynamics.^[^ ^33^ ^]^ High MTFR2 expression enhances mitochondrial fission, increasing the pool of transferable mitochondria via TNTs. This coordinated transfer of FAs and mitochondria establishes a metabolic synergy that sustains HCC cell survival under nutrient stress.

The identification of MTFR2 as a pivotal regulator of mitochondrial dynamics positions it as a promising target for HCC therapy. Our findings establish MTFR2 in aHSCs as a key driver of HCC progression, orchestrating mitochondrial fission and the transfer of metabolic resources to tumor cells. Within the TME, HSC activation elevates MTFR2 expression, creating a stromal support system that sustains tumor growth through FA and mitochondrial provision. Targeting MTFR2‐mediated mitochondrial dynamics could disrupt this support, potentially slowing HCC progression and improving patient outcomes. Future research should prioritize the development of selective MTFR2 inhibitors, possibly combined with therapies that dismantle stromal metabolic contributions. In addition, further elucidation of the MTFR2‐DRP1 relationship could uncover novel mechanistic insights, enhancing therapeutic precision. By linking our findings to the broader literature on mitochondrial dynamics and tumor metabolism, this study provides a foundation for exploring MTFR2‐targeted strategies in HCC management.

## 4 Experimental Section

### Human Tissues

Matched fresh tumor tissues, margin tumor tissues (1 cm‐wide zones centered on the tumor border), and para‐tumor tissues (at least 2 cm from the tumor border) from six primary HCC patients were collected at the First Affiliated Hospital of Chongqing Medical University. This study was conducted in accordance with both the Declarations of Helsinki and Istanbul. All participants provided informed consent, and the collection procedures were approved by the Institutional Review Board of the First Affiliated of Chongqing Medical University (Approval No. K2023‐440, Chongqing, China).

### Mice and Tumor Model

The research was approved by the Ethics Committee of Chongqing Medical University (Approval No. IACUC‐CQMU‐2024‐0232, Chongqing, China). All animal experiments followed the Guidelines for the Care and Use of Laboratory Animals. Male BALB/c‐nu nude mice were purchased from Enswell Biotechnology Co., Ltd. (Chongqing, China) and were implanted with liver tumors at 6–8 weeks of age. For orthotopic tumor model, MHCC‐97H cells (1 × 10^6^ cells) were resuspended in 20 µL Ceturegel Matrix LDEV‐Free Matrigel (Yeasen, Catalog No. 40183ES10) and injected to the left lateral liver lobe after the mice was anesthetized with Delivectorrm Avertin (Catalog No. DW3106), obtained from Dowobio (Shanghai, China). For subcutaneous tumor formation, mice were injected subcutaneously with MHCC‐97H cells (1 × 10^6^ cells in 100 µL matrigel) or MHCC‐97H cells plus WT or KD LX2 cells (2 × 10^5^ cells). Tumors were analyzed four weeks later.

### Cell Culture

The human hepatocellular carcinoma cell line Huh7 and the human hepatic stellate cell line LX2 were obtained from Sunncell Biotech (Wuhan, China). The human hepatocellular carcinoma cell line MHCC‐97H was kindly provided by Dr. Fang Ren (Chongqing Key Laboratory of Sichuan‐Chongqing Co‐construction for Diagnosis and Treatment of Infectious Diseases Integrated Traditional Chinese and Western Medicine, Chongqing Hospital of Traditional Chinese Medicine, Chongqing, China). All cell lines were maintained in DMEM containing 10% fetal bovine serum (FBS) and incubated at 37 °C in a humidified incubator with 5% CO_2_.

### Small Interfering RNA (siRNA), Plasmids, Lentiviruses, and Transfection

Scrambled small interfering RNA (siNC) and siRNA targeting MTFR2 (siMTFR2) were purchased from Tsingke Biotech Co., Ltd. (Beijing, China), and the sequences are shown in Table S1 (Supporting Information). The DRP1 overexpression plasmid (pCMV‐DNM1L(human)‐3Flag‐NEO(OE)) was synthesized by Hunan LeapWal Biotech Co., Ltd. (Hunan, China), whose sequence is shown in Table S2 (Supporting Information). Transfections of both siRNA and overexpression plasmid were carried out using Lipo8000 Transfection Reagent (Beyotime, Catalog No. C0533) for 48 h, based on the manufacturer's protocol. For the stable knockdown of MTFR2 in LX2 cells, short hairpin RNAs (shRNAs) targeting MTFR2 was constructed in the shRNA expression vector (System Biosciences, CA), three target sequences were synthesized and inserted into the vector by Hunan LeapWal Biotech Co., Ltd. (Hunan, China), listed in Table S3 (Supporting Information). shRNA with a nontargeting sequence was utilized as a negative control. To establish a fluorescent tracking mitochondria cell line, pLVW‐CMV‐mito‐mCherry‐EF1‐Puro was also generated and titrated from Hunan LeapWal Biotech Co., Ltd.

### Chemicals and Antibodies

Reagents including tyrphostin A9 (TA9) (Catalog No. HY‐15511), MG‐132 (Catalog No. HY‐13259), TMZ (Catalog No. HY‐B0968), and CQ (Catalog No. HY‐17589A) were purchased from MedChem Express (Monmouth Junction, NJ, USA). NTL (Catalog No. T1327) were obtained from Topscience Co., Ltd. (Shanghai, China). L‐778123 hydrochloride (Catalog No. L880006) was purchased from Macklin Biochemical Co., Ltd. (Shanghai, China). CHX (Catalog No. A10036) was obtained from Adooq Bioscience LLC. (Irvine, CA). Antibodies used for western blot, immunohistochemistry (IHC), and immunofluorescence: anti‐collagen type I (COL1) polyclonal antibody (Catalog No. 14695‐1‐AP) was purchased from Proteintech Group, Inc. (Chicago, USA) and anti‐alpha smooth muscle actin (α‐SMA) mouse monoclonal antibody (Catalog No. BM0002) was purchased from Boster Biological Co. Ltd. (Wuhan, China). Antibodies used for western blot: anti‐WNT5A/B rabbit polyclonal antibody (Catalog No. 55184‐1‐AP) and anti‐MIRO1 rabbit polyclonal antibody (Catalog No. 21560‐1‐AP) were obtained from Proteintech Group, Inc. (Chicago, USA), and anti‐RAC1 rabbit monoclonal antibody (Catalog No. HA722225), anti‐ATP8A1 rabbit polyclonal antibody (Catalog No. ER1902‐61), anti‐SDHB recombinant rabbit monoclonal antibody (Catalog No. ET1706‐30), anti‐UQCRC2 rabbit polyclonal antibody (Catalog No. ER1803‐08), anti‐MTCO1 rabbit polyclonal antibody (Catalog No. HA500517), anti‐NDUFB8 recombinant rabbit monoclonal antibody and anti‐CPT2 recombinant rabbit monoclonal antibody (Catalog No. ET1611‐64), and Phospho‐Histone H2A.X (S139) recombinant rabbit monoclonal antibody (Catalog No. ET1602‐2) were purchased from HuaBio Biotechnology (Hangzhou, China). Antibodies used for western blot and immunofluorescence: anti‐DRP1 mouse antibody (Catalog No. 221099) was bought from ZEN‐BIOSCIENCE (Chengdu, China), anti‐MTFR2 rabbit polyclonal antibody (Catalog No. 26569‐1‐AP) was obtained from Proteintech Group, Inc. (Chicago, USA), anti‐CPT1A rabbit polyclonal antibody (Catalog No. AF6558) was obtained from Beyotime Institute of Biotechnology (Shanghai, China), and anti‐ACC1 monoclonal antibody (Catalog No. 67373‐1‐Ig) was obtained from Proteintech Group, Inc. (Chicago, USA). Antibodies used for immunofluorescence: anti‐TOMM20 mouse monoclonal antibody (Catalog No. 66777‐1‐Ig) was obtained from Proteintech Group, Inc. (Chicago, USA), anti‐Histone H3 mouse monoclonal antibody (Catalog No. EM30605) and iFluor 647 conjugated anti‐GFAP mouse monoclonal antibody (Catalog No. HA600103F) were bought from HuaBio Biotechnology (Hangzhou, China).

### Histological and Immunohistochemical Analysis

Tissue sections were fixed in 4% paraformaldehyde, embedded in paraffin, and cut into 5‐µm‐thick sections. Sections were stained with hematoxylin for 5 min, followed by eosin for 2 min. For IHC, tissue sections were deparaffinized, rehydrated, and subjected to antigen retrieval by heating in a citrate buffer (pH 6.0) for 20 min. The sections were then blocked with 5% bovine serum albumin (BSA) for 30 min at room temperature to minimize nonspecific binding. The sections were incubated with the primary antibodies overnight at 4 °C, followed by incubation with an HRP‐conjugated secondary antibody for 1 h at room temperature. Detection was achieved using 3,3′‐diaminobenzidine (DAB), and sections were counterstained with hematoxylin for nuclei visualization. As for IF staining, tissue sections and cell coverslips, fixed in 4% paraformaldehyde for 15 min, were permeabilized with 0.4% Triton X‐100 for 10 min, and blocked with 5% BSA for 1 h. Primary antibodies were incubated overnight at 4 °C, followed by fluorophore‐conjugated secondary antibodies for 1 h at room temperature in the dark. Actin‐Tracker Red‐Rhodamine (Beyotime, Catalog No. C2207S) was used to stain skeleton as described in protocol. Mounting medium with DAPI (Abcam, Catalog No. ab104139) was used to stain nuclei and mount the section. Samples were imaged using a confocal laser scanning microscopy (CLSM, Leica TCS SP8, Mannheim, Germany). 3D rendering was performed in Imaris Viewer 10.2.0. Regarding multiplex immunofluorescence, a tyramide signal amplification approach was performed on tissue slides as previously described.^[^ ^34^ ^]^

### Activation of the Hepatic Stellate Cells

Huh7 or MHCC‐97H cells were cultured in medium using the same number of cells and amount of medium for 48 h. Tumor conditioned medium (TCM) was prepared by collecting the medium when cells reached 70%–90% confluence, filtered with a 20 µm pore filter and mixed with fresh media at 8:2 ratio. Complete medium was used as a control. HSCs were seeded at a density of 5 × 10^5^ cells per 100 mm^2^ dish and treated with 5 mL of TCM for 72 h without changing the media.

### Evaluating the Morphology of the Mitochondria

Liver tissue samples from HCC and adjacent nontumor regions were fixed, embedded, and sectioned for immunoelectron microscopy. Sections were labeled with anti‐GFAP antibodies followed by gold‐conjugated secondary antibodies to identify HSCs. Mitochondrial morphology was visualized with a transmission electron microscope (JEM‐1400FLASH, JEOL, Japan) after staining with uranyl acetate and lead citrate. For assessing mitochondria shape in vitro, mitochondria were isolated following the manufacturer's instructions (Beyotime, Catalog No. C3601) and detected by flow cytometry. At the same time, the images of IF staining for TOMM20 were obtained and the size of mitochondria was analyzed using FIJI‐ImageJ software (National Institutes of Health, DC, USA) and classified according to the method described in previous report.^[^ ^35^ ^]^

### Differential Expressed Analysis of Mitochondrial Dynamics‐Related Genes

The GSE68001, GSE39469, GSE680000, GSE11954, and GSE52234 datasets were used from the GEO public database to dig out the key genes. Twenty‐three mitochondria dynamics genes were identified from MitoCarta 3.0,^[^ ^36^ ^]^ presented in Table S4 (Supporting Information). The “limma” R package was conducted to figure out differentially expressed genes of mitochondrial dynamic genes between HSC and aHSC, and visualized using a volcano plot created with the ggplot2 package in R, highlighting differentially expressed genes (threshold: *p*‐value < 0.05 and absolute log2 fold‐change > 1).

### Protein Extraction and Immunoblotting

Tissue and cell samples were lysed in RIPA buffer (Beyotime, Catalog No. P0013B) with PMSF (Beyotime, Catalog No. ST506). After lysis, samples were centrifuged at 12000× *g* for 15 min at 4 °C, and protein concentrations were determined using a BCA Protein Assay Kit (GlpBio, Catalog No. GK10009), monitored by enhanced chemiluminescent (ECL, AP34L014, Life‐iLab, China). For Western blotting, equal protein amounts were resolved by SDS‐PAGE and transferred to PVDF membranes. Membranes were blocked with 5% skim milk in TBST, incubated with primary antibodies overnight at 4 °C, followed by HRP‐conjugated secondary antibodies. Protein bands were visualized using ECL (BIO‐OI, China) and quantified using ImageJ.

### Measurement of Oxygen Consumption Rate

aHSCs under different treatments were seeded in a 24‐well plate for Seahorse measurements (Seahorse XFe24 FluxPaks, Agilent, Catalog No. 102340‐100). Mitochondrial respiration was measured with a modified protocol.^[^ ^37^ ^]^ Data were analyzed using Wave for Desktop (Agilent Technologies).

### Cell Coculture System

HCC cells were first labeled with 1 × 10^−6^ m CellTrace green (CFDA SE, Yeasen, Catalog No. 40714ES76) by incubating in serum‐free medium at 37 °C under 5% CO_2_ for 30 min according to the manufacturer's protocols. For FA transfer assays, HSCs were preloaded with 1 × 10^−3^ m Red C12 (BODIPY 558/568 C12, MCE, Catalog No. HY‐138226) in CM for 16 h, then rigorously washed three times with CM and equilibrated for 1 h to remove extracellular dye aggregates. Mitochondrial transfer assays involved labeling donor HSCs with Mito‐Tracker Far Red (Beyotime, Catalog No. C1032) in CM for 30 min, followed by three washes and a 1‐h incubation to eliminate unbound probes. Labeled HCC cells (CellTrace green positive) and HSCs (Red C12 positive or MitoTracker positive) were then cocultured in HBSS at a 1:1 ratio for defined times after cell adhesion. Post‐coculture, HCC cells were isolated via fluorescence‐activated cell sorting (FACSAria III, BD Biosciences) using a 488 nm laser, achieving >98% purity. The intercellular transfer efficacy of FAs (Red C12) and mitochondria (MitoTracker Far Red, Beyotime, Catalog No. C1032) was quantitatively assessed through integrated flow cytometric and confocal imaging approaches. The intensity of Red C12 or MitoTracker Far Red in CellTrace Green‐positive HCC cells was analyzed using a CytoFLEX LX flow cytometer (Beckman Coulter) to determine fluorescence intensity distributions across three biological replicates. Parallel confocal quantification utilized z‐stack imaging (Leica TCS SP8, Mannheim, Germany) processed through LAS Application Suite X 3.7.2 software, where the mean fluorescence intensity of transferred cargos in recipient HCC cells was calculated from six biological replicates (≥50 cells per replicate).

### Cell Viability and Apoptosis Assays

HCC cells sorted out using flowcytometry were seeded in 96‐well plates at a density of 5 × 10^3^ cells per well. and allowed to adhere for 4–6 h. After attachment, 10 µL of CCK‐8 solution (TargetMol, Catalog No. C0005) was added to each well and incubated for 2 h at 37 °C. Absorbance was then measured at 450 nm to assess cell viability using a microplate reader (Tecan Group Ltd., Switzerland). CellTrace green‐labeled cancer cells cocultured with HSC were harvested (including the medium), washed twice with cold PBS, incubated with Annexin V‐APC and DAPI for 15 min in the dark at room temperature, followed by measuring with a flow cytometer (CytoFLEX, BeckmanCoulter, USA). Total apoptosis rate was calculated as combination of (Annexin V positive/DAPI positive, late apoptosis) and (Annexin V positive/DAPI negative, early apoptosis) percentages.

### Total FAs Profiling in Cancer Cells Using UPLC‐MS/MS

The total FAs profile acquisition instrument system mainly includes ultra performance liquid chromatography (UPLC) (ExionLC AD) and tandem mass spectrometry (MS/MS) (QTRAP6500+). Hydrogen served as the carrier gas. FAs were detected in selected ion monitoring mode and normalized against internal standards based on the metware database. Data processing was carried out using Analyst 1.6.3 software.

### Fluorescent FAs Pulse‐Chase Experiment

HSCs were incubated with CM containing 1 × 10^−3^ m Red C12 for 16 h. Cells were then washed three times with CM, incubated for 1 h to allow the fluorescent lipids to incorporate into LDs or cellular membranes, and then chased for the time indicated in CM or TCM in the absence or presence of various drugs. Mitochondria were labeled with 100 × 10^−9^ m MitoTracker Deep Far Red for 30 min prior to imaging. To label LDs, 2 × 10^−3^ m BODIPY 493/503 (MCE, Catalog No. HY‐W090090) was added to HSCs at immediately prior to imaging.

### Scanning Electron Microscopy

Cells were seeded on 12‐mm glass coverslips and fixed using 2.5% glutaraldehyde in 0.1 m sodium cacodylate buffer. Add 0.1 mol L^−1^ PBS to the wells with the samples, ensuring they are submerged, and gently shake in one direction. Wash twice for 5 min each. After washing, remove the PBS and add 3% glutaraldehyde for fixation at 4 °C for 1 h. Then, wash the samples three times with ultrapure water, 10 min each. Post‐fixation, treat the samples with 1% osmium tetroxide for 1 h, followed by three additional washes with ultrapure water. Dehydrate the samples with a graded ethanol series (30%–100%), 15 min per step. Once dehydrated, dry the samples using a critical point dryer to prevent deformation. Mount the dried samples onto stubs using conductive adhesive and sputter‐coat them. Finally, observe the samples using a JSM‐IT700HR (JEOL, Japan) scanning electron microscope.

### Tumor Spheroid Generation and Drug Treatment

HCC cells and LX2 cell were mixed at a ratio of 4:1 and resuspend in mixture of Ceturegel Matrix LDEV‐Free Matrigel (Yeasen, Catalog No. 40183ES10) and CM (7:3). A total of 2 × 10^3^ cells (20 µL) were seeded in 24‐well plate and gelled in a cell culture incubator for at least 40 min. The plates were incubated in CM for 6 d at 37 °C in a humidified atmosphere of 5% CO_2_. For drug treatment, the cells were seeded with or without L778123 (10 × 10^−6^ m) and NTL (10 × 10^−6^ m) in PBS for additional 4 d. The spheroid volume was calculated using the formula: (*L* × *I* ^2^)/2, where *L* represents the long diameter and *I* represents the short diameter of the spheroid.

### Cell Live/Death Detection in Spheroid

Spheroid cell live/death was detected using the Beyo3D Calcein AM Staining Solution (Beyotime, Catalog No. C1367S) and Beyo3D PI Staining Solution (Beyotime, Catalog No. C1352S). Spheroids were incubated in 10 µL Calcein AM staining solution in 1 mL PI staining solution for 10 min in a 37 °C incubator and images were obtained using the CLSM. The green fluorescence (Calcein AM) for live cell viability was quantified and expressed as a percentage of the total fluorescence (red and green).

### Measurement of Intracellular Oxidative Stress

Huh7 or MHCC‐97H cells, labeled with 1 × 10^−6^ m CellTrace Green and treated with 10 × 10^−6^ m NTL, were co‐cultured with L778123‐pretreated LX2 cells in six‐well plates for 24 h. To assess ROS (O_₂_^•−^) levels, cells were then incubated with 25 × 10^−6^ m DHE (Beyotime, Catalog No. S0063) at 37 °C for 25 min in the dark. After incubation, the DHE‐containing medium was removed, and cells were washed with PBS. DHE fluorescence intensity in CellTrace Green‐positive HCC cells was quantified by flow cytometry.

### RNA Extraction and Quantitative RT‐PCR

Total RNA was extracted using RNA‐QUICK Purification Kit (Catalog No. ES‐RN001) purchased from YISHAN Biochemical Co., Ltd. (Shanghai, China). Reverse transcription was performed using M‐MLV reverse transcriptase (Accurate Biology, Catalog No. AG11728). Real‐time PCR was performed with SYBR Green Pro Taq HS (Accurate Biology, Catalog No. AG11759) following the manufacturer's protocol. The mRNA levels were calculated using the ΔCt method and normalized to β‐actin. Primers for human genes encoding MTFR2, DRP1, and β‐actin were obtained from Sangon Biotech Co. Ltd. (Shanghai, China). All primers used in this study are listed in Table S5 (Supporting Information).

### Cycloheximide Chase Assay

The half‐life of DRP1 was assessed using a CHX chase assay. Approximately 3 × 10^5^ cells were seeded in 60 mm dishes and cultured for 24 h. Cells were then treated with CHX (10 µg mL^−1^) for 0, 4, 8, and 12 h. At each time point, cells were collected, and total protein lysates were prepared for western blot analysis to detect DRP1.

### Co‐Immunoprecipitation (Co‐IP)

BeaverBeads Protein A/G kit (Beaverbio, Catalog No. 22202‐20), with magnetic beads coupled to A/G protein, was used for co‐IP. We rinsed 50 µL of magnetic beads and incubated them with antibodies (1 µg of Rabbit Control IgG (Abclonal, Catalog No. AC005) or anti‐MTFR2 antibody (Proteintech, Catalog No. 26569‐1‐AP)) at 4 °C overnight. 500 µg of total protein extract was mixed with the antibody–bead complex, and the mixture was incubated at 4 °C overnight. The complex was rinsed and the proteins eluted by boiling the beads in SDS‐PAGE loading buffer at 95 °C for 5 min. Samples were obtained after centrifuging at 13000 *g* for 10 min and analyzed by western blot.

### Statistical Analyses

Statistical analyses were conducted using GraphPad Prism 10.0 (GraphPad Software Inc.). The Shapiro–Wilk test was applied to assess data normality prior to performing *t*‐tests, one‐way ANOVA, or Pearson correlation analyses. One‐way ANOVA was used to compare the means of more than two groups, while Student's *t*‐test compared the mean values of two groups. The χ^2^ test analyzed the association between plasma EPI levels and clinical or pathological characteristics in patients with breast cancer. Pearson correlation was used to determine the relationship between two variables. Results are reported as mean ± SD from three or more independent experiments.

## Conflict of Interest

The authors declare no conflict of interest.

## Author Contributions

L.Z., B.Z., and J.Y. contributed equally to this work. L.Z. developed the study concept; Z.W. and N.J. supervised the project. Z.L. performed the computational analysis; L.Z., B.Z., C.R., and Z.H. conducted experiments; L.Z. and Q.L. collected the clinical data; J.L. and N. J. contributed biopsy samples and pathology analysis. L.Z. and J.Y. performed the data analysis. L.Z., Z.H., and N.J. wrote the manuscript with the help of Z.W. All authors read and approved the final version of the manuscript.

## Supporting information



Supporting Information

## Data Availability

The data that support the findings of this study are available from the corresponding author upon reasonable request.
