# Supplementary material for: MTFR2‐Mediated Fission Drives Fatty Acid and Mitochondrial Co‐Transfer from Hepatic Stellate Cells to Tumor Cells Fueling Oncogenesis
Source: Adv Sci (Weinh). 2025 May 14;12(23):2416419. doi: 10.1002/advs.202416419 (PMC12199435; doi:10.1002/advs.202416419)
Supplement: Supplementary file 1 — Supporting Information [file ADVS-12-2416419-s001.pdf]

ADVANCED SCIENCE
Open Access

Supporting Information

for *Adv. Sci.*, DOI 10.1002/advs.202416419

MTFR2-Mediated Fission Drives Fatty Acid and Mitochondrial Co-Transfer from Hepatic Stellate Cells to Tumor Cells Fueling Oncogenesis

La Zhang, Baoyong Zhou, Jun Yang, Cong Ren, Jing Luo, Zhenghang Li, Qiang Liu, Zuotian Huang, Zhongjun Wu* and Ning Jiang**

Supporting Information

MTFR2-Mediated Fission Drives Fatty Acid and Mitochondrial Co-Transfer from Hepatic Stellate Cells to Tumor Cells Fueling Oncogenesis

La Zhang, Baoyong Zhou, Jun Yang, Cong Ren, Jing Luo, Zhenghang Li, Qiang Liu, Zuotian Huang, Zhongjun Wu*, Ning Jiang**

L. Zhang, Z. Li, Q. Liu, Z. Huang, Z. Wu

Department of Hepatobiliary Surgery

The First Affiliated Hospital of Chongqing Medical University

College of Basic Medical Sciences of Chongqing Medical University

Chongqing 400016, China

Email: huangzuotian@cqu.edu.cn; wzjtcy@126.com

L. Zhang, J. Luo, N. Jiang

Department of Pathology

College of Basic Medical Sciences

Chongqing Medical University

Chongqing 400016, China

Email: jiangning@cqmu.edu.cn

L. Zhang, J. Luo, N. Jiang

Molecular Medicine Diagnostic and Testing Center

Chongqing Medical University

Chongqing 400016, China

L. Zhang, J. Luo, N. Jiang

Department of Pathology

The First Affiliated Hospital of Chongqing Medical University

Chongqing 400016, China

B. Zhou

Department of Hepatobiliary Surgery

Bishan Hospital of Chongqing Medical University

Chongqing 400016, China

J. Yang

Department of Anesthesiology

The First Affiliated Hospital of Chongqing Medical University

Chongqing 400016, China

C. Ren

Department of Medicinal Chemistry College of Pharmacy

Chongqing Medical University

Chongqing 400016, China

N. Jiang

Department of Biochemistry and Molecular Biology

Chongqing Medical University

Chongqing 400016, China

Figure S1

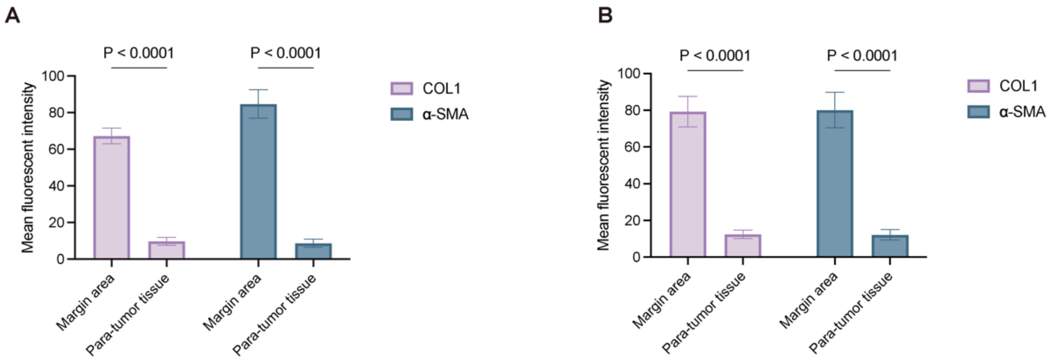

Figure S1. Mean fluorescence intensity of α-SMA and COL1 in the tumor margin and para-tumor tissue in human HCC tumor regions (A), and xenografted mouse models (B). Data are represented as mean ± SD, n = 6, Two-way ANOVA was performed).

Figure S2

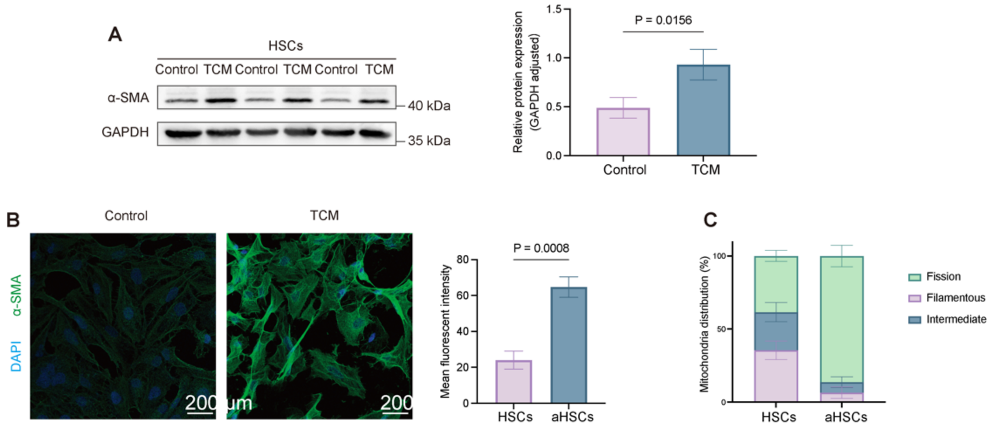

Figure S2. Hepatic stellate cells (HSCs) activated by tumor-conditioned medium (TCM). A) Western blot and quantification show the protein expression of α-SMA in hepatic stellate cells (HSCs) after TCM treatment (Data are represented as mean ± SD, n = 3, One-way ANOVA was performed). B) Immunofluorescence (IF) staining and mean fluorescent intensity of α-SMA in HSCs (Data are represented as mean ± SD, n = 3, One-way ANOVA was performed). Scale bars: 200 μm. C) Classification of cells based on percentage distribution of filamentous, intermediate and fission mitochondrial morphology in HSCs and aHSCs. n = 10, each group (Data are represented as mean ± SD).

Figure S3

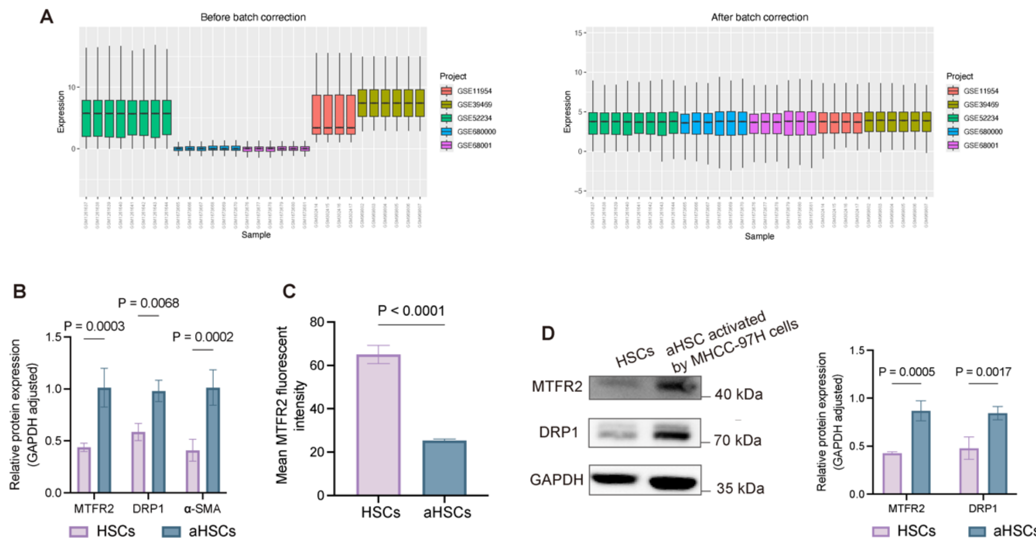

Figure S3. MTFR2 was upregulated in activated hepatic stellate cells (aHSCs). A) Gene expression before and after batch effect correction. B) Quantification of the expression level of MTFR2, DRP1 and α-SMA in HSCs and aHSCs (treated by TCM from Huh7 cells), corresponding to Figure 1J (Data are represented as mean ± SD, n = 3, 2-way ANOVA was performed). C) The mean fluorescent intensity of MTFR2 in HSCs and aHSCs (treated by TCM from Huh7 cells), corresponding to Figure 1K. (Data are represented as mean ± SD, n = 3, One-way ANOVA was performed). D) The protein level of MTFR2 and DRP1 in HSCs cultured in TCM from MHCC-97H cells. (Data are represented as mean ± SD, n = 3, Two-way ANOVA was performed).

Figure S4

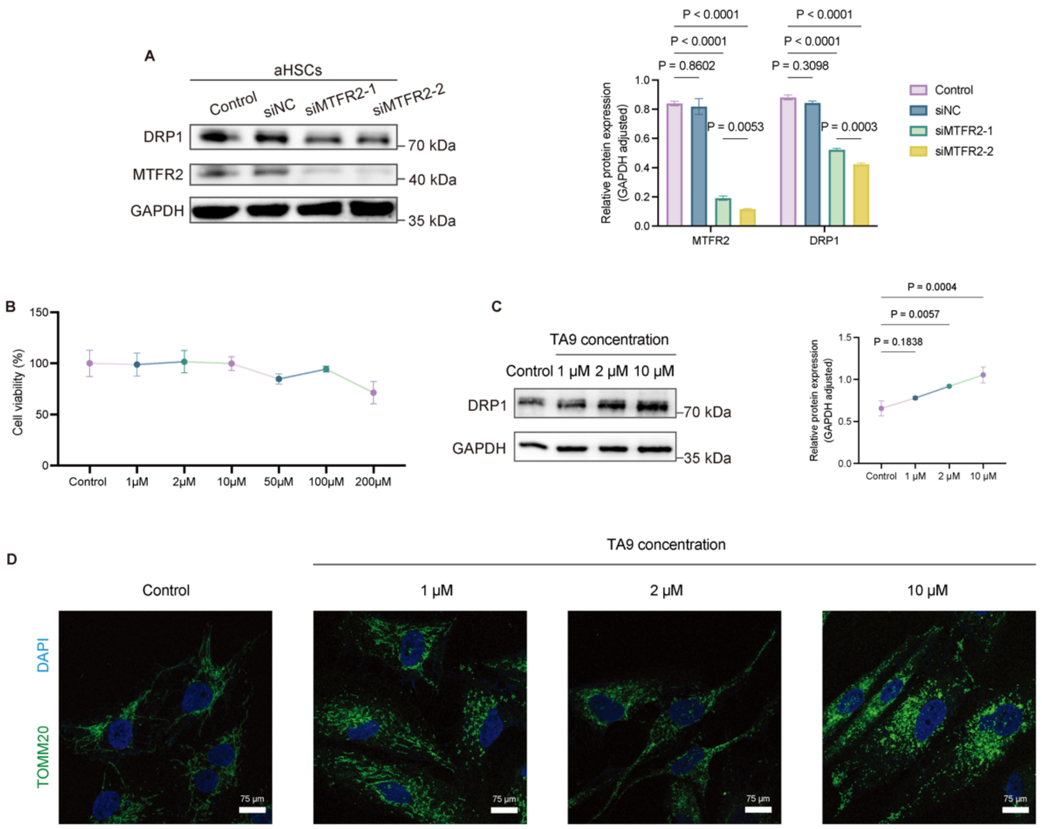

Figure S4. siRNA targeting MTFR2 screening and concentration of tyrphostin A9 (TA9) choosing. A) Western blot shows the protein level after transfected with different siRNAs and the quantification based on GAPDH (n = 3). siMTFR2-2 was used in the following experiment (Data are represented as mean ± SD, n = 3, Two-way ANOVA was performed). B) Cell viability of aHSCs treated with different concentration of TA9 (Data are represented as mean ± SD, n = 4). C) Western blot reveals the protein expression level of DRP1 in aHSCs treated with TA9 and quantification based on GAPDH (Data are represented as mean ± SD, n = 3, One-way ANOVA was performed). D) Immunofluorescence (IF) staining of TOMM20 suggests the morphology of mitochondria from aHSCs. Scar bars: 75 µm. 10 µM was used in the following experiments.

Figure S5

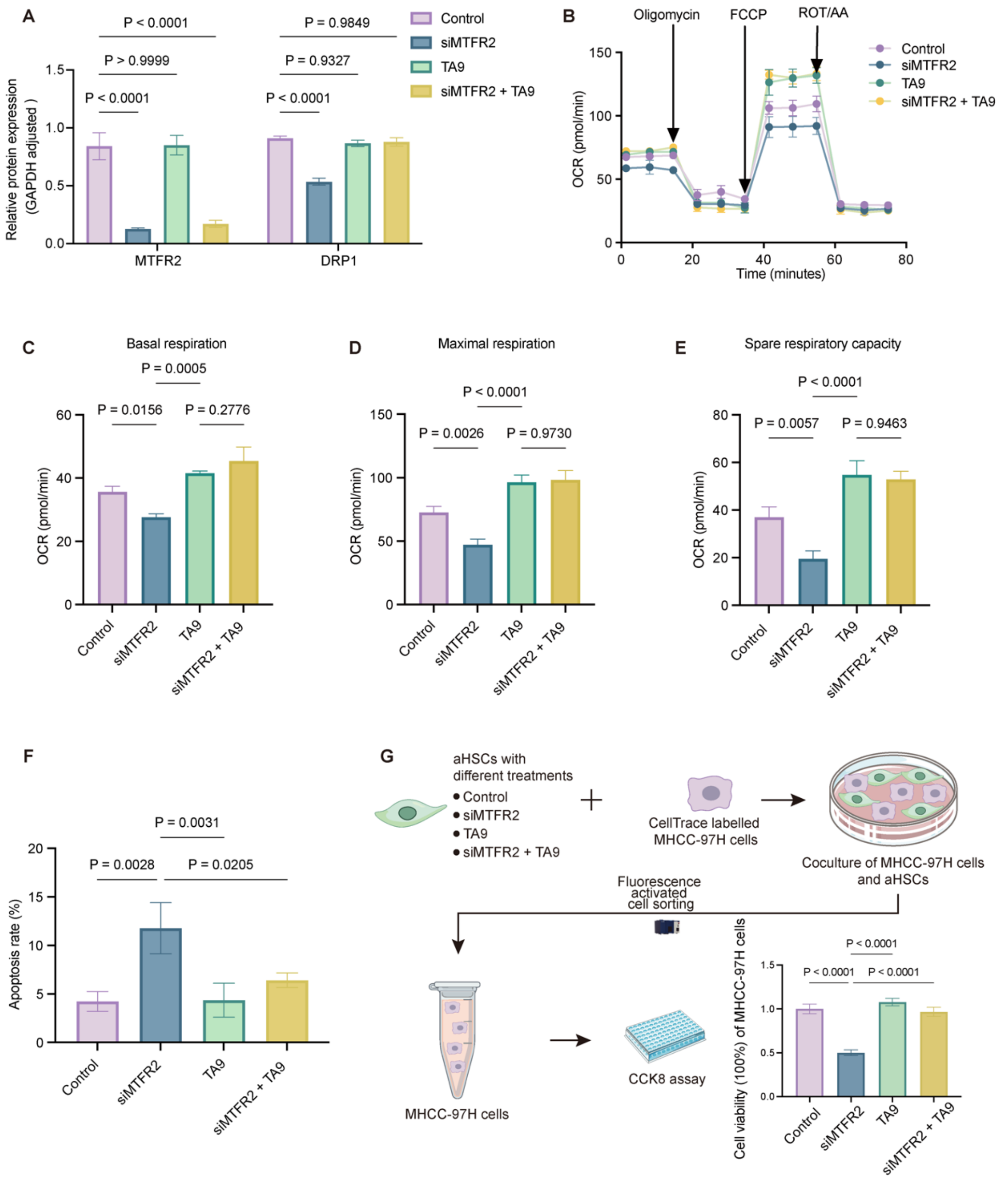

Figure S5. The expression of MTFR2 in HSCs influence HCC cancer survival status. A) Quantification of western blot analysis for protein expression in Figure1L (Data are represented as mean ± SD, n = 3, Two-way ANOVA was performed). B-E) OCR analysis of aHSCs under different treatment using the Mito Stress kit for Seahorse instruments. Basal and maximal respiration rates are shown in C and D, as well as spare respiratory capacity was shown in E (Data are represented as mean ± SD, n = 3, Two-way ANOVA was performed). F) Quantification of Huh7 cells' apoptosis rate in Figure

1Q. (Data are represented as mean ± SD, n = 3, Two-way ANOVA was performed). G) The CCK8 assay measured the proliferation rate of MHCC-97H cells coculturing with aHSCs processed various procedures sorted by flow cytometry (Data are represented as mean ± SD, n = 4, One-way ANOVA was performed).

Figure S6

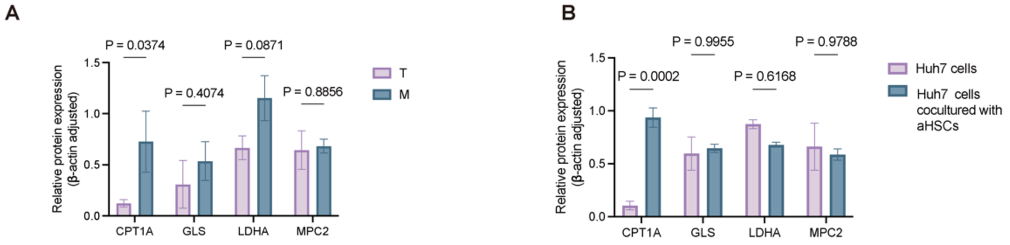

Figure S6. Quantification of protein related to different energy metabolic enzymes analyzed by western bolt. A) The relative protein expression levels extracted from tumor tissue and margin area from HCC patients (Data are represented as mean ± SD, n = 3, Two-way ANOVA was performed). B) Quantification of protein expression from Huh7 cells cultured alone and Huh7 cells cocultured with aHSCs (Data are represented as mean ± SD, n = 3, Two-way ANOVA was performed).

Figure S7

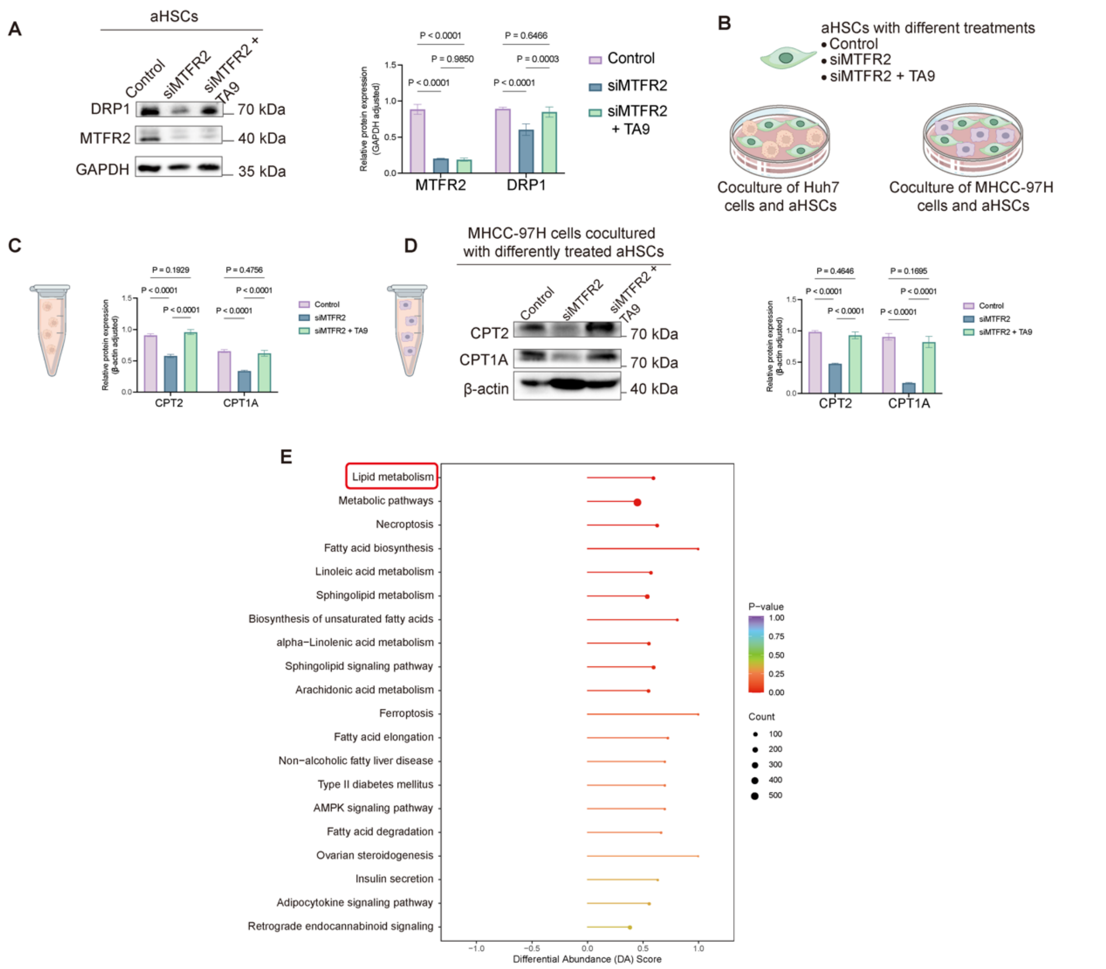

Figure S7. Modulating MTFR2 and mitochondrial shape of aHSCs influence the extent of FAO of HCC cells. A) Western blot analysis demonstrating the efficacy of interfering MTFR2 and DRP1 expression and quantification based on GAPDH (Data are represented as mean ± SD, n = 3, Two-way ANOVA was performed). B) Illustration of coculture HCC cells with aHSCs in different treatment. C) Quantification of CPT1A and CPT2 expression level analyzed by western blot (Figure 2E) in Huh7 cells cocultured with different treated aHSCs (control, siMTFR2, and siMTFR2 + TA9) (Data are represented as mean ± SD, n = 3, Two-way ANOVA was performed). D) CPT1A and CPT2 expression and quantification in MHCC-97H cells cocultured with aHSCs with treatments suggested as (B) (Data are represented as mean ± SD, n = 3, Two-way ANOVA was performed). E) Kyoto Encyclopedia of Genes and Genomes (KEGG) pathway enrichment analysis of differential expressed lipids from Huh7 cells

cocultured with aHSCs (siMTFR2 + TA9) vs. Huh7 cells cocultured with aHSCs (siMTFR2) (n = 5).

Figure S8

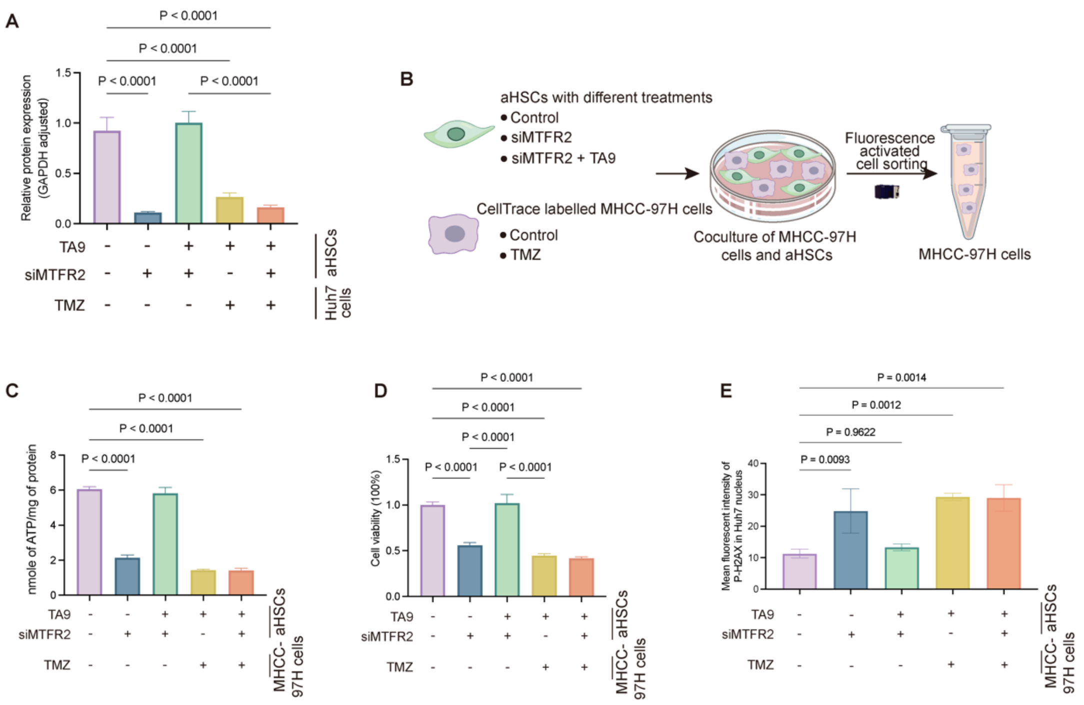

Figure S8. Modulating MTFR2 and mitochondrial shape of aHSCs influence the energy production pathway and proliferation of HCC cells. A) Quantification of CPT1A expression level in Huh7 cells (Figure 1I) based on GAPDH, corresponding to Figure 2E (Data are represented as mean ± SD, n = 3, One-way ANOVA was performed). B) Schematic representation of the experimental setup showing aHSCs treated with either control, siMTFR2, or a combination of siMTFR2 and TA9, followed by coculture with CellTrace-labeled MHCC-97H cells. Fluorescence-activated cell sorting was employed to obtain MHCC-97H cells. C) Quantification of ATP levels expressed as nmol ATP/mg of protein in MHCC-97H cells under different treatment conditions as shown in (B) (Data are represented as mean ± SD, n = 3, One-way ANOVA was performed). D) Assessment of cell viability in MHCC-97H cells cocultured with aHSCs following treatment with TA9, siMTFR2, and TMZ (Data are represented as mean ± SD, n = 4, One-way ANOVA was performed). (E) Mean fluorescent intensity of P-H2AX in Huh7 cell (CellTrace labeled) nucleus (Data are represented as mean ± SD, n = 3, at least 50 Huh7 cells analyzed per replicate, One-way ANOVA was performed).

Figure S9

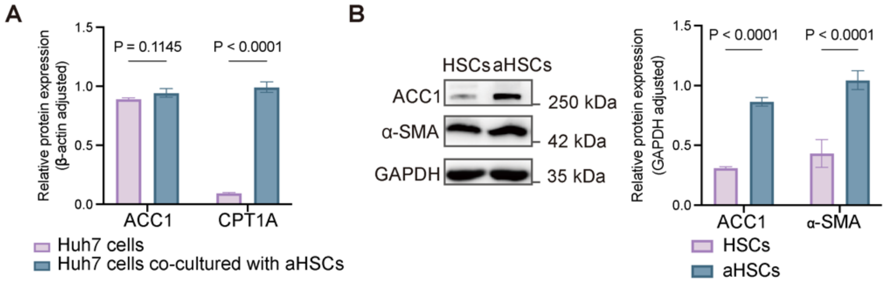

Figure S9. Western blot analysis. A) Western blot quantification the ACC1 and CPT1A level of Huh7 cells and Huh7 cells cocultured with aHSCs (Figure 3A, Data are represented as mean ± SD, n = 3, Two-way ANOVA was performed). B) Western blot analysis of ACC1 and α-SMA expression of HSCs compared with aHSCs and its quantification is shown in the right panel (Data are represented as mean ± SD, n = 3, Two-way ANOVA was performed).

Figure S10

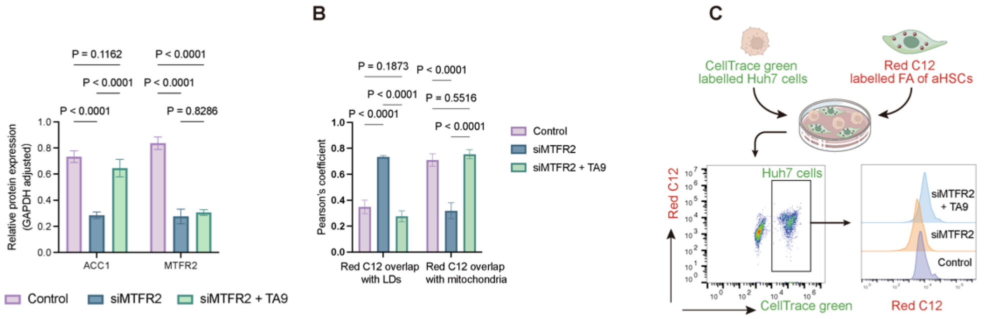

Figure S10. A) Quantification of the protein expression level of ACC1 corresponding to Figure 3G (Data are represented as mean ± SD, n = 3, Two-way ANOVA was performed). B) Relative cellular localization of Red C12 was quantified by Pearson's coefficient analysis (Data are represented as mean ± SD, n = 3, Two-way ANOVA was performed). C) Flow cytometry analysis the FA transfer from aHSCs with different treatment to Huh7 cells.

Figure S11

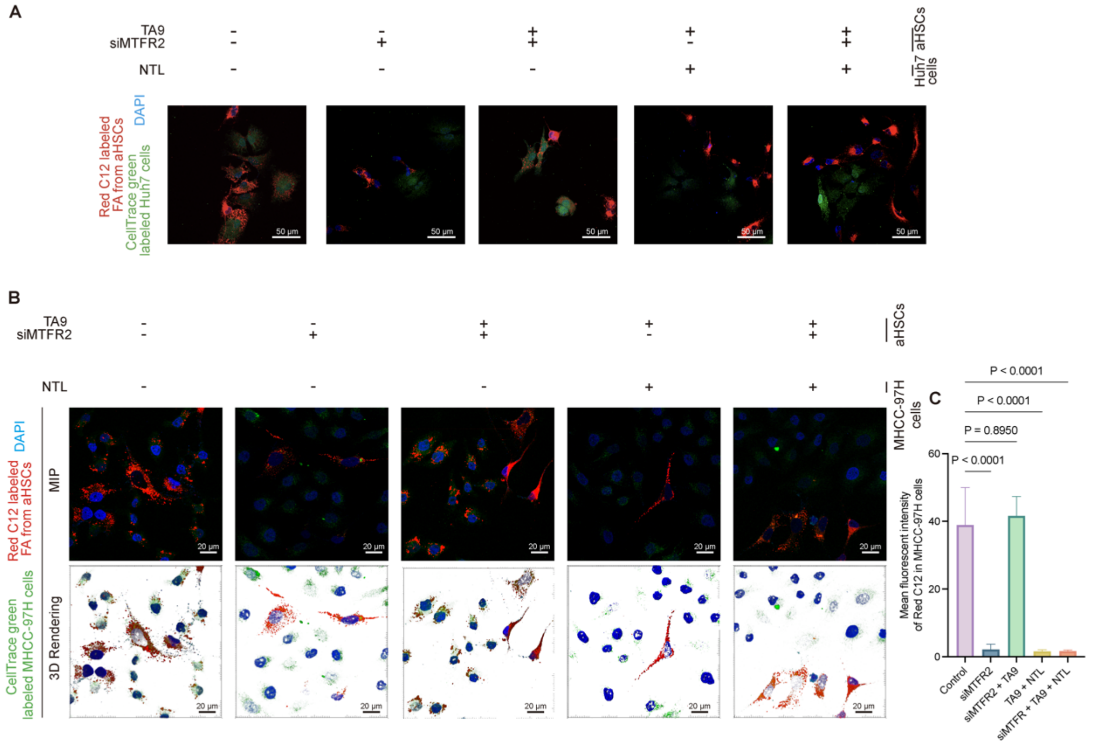

Figure S11. MTFR2 promoted the transfer of fatty acids (FA) to HCC cells. A) Maximum intensity projection (MIP) corresponding to the 3D-reconstructed confocal immunofluorescence (IF) images (Figure 3K) demonstrating atty acids (FA) transfer dynamics from HSCs (Red C12-labeled FA) and Huh7 cells (CellTrace green-labeled) under differential treatment conditions. The MIP algorithm was applied to Z-stack acquisitions to optimize visualization of intercellular FA trafficking. Scale bar: 50 µm. B and C) IF images (both MIP and 3d reconstruction by Imaris) of Red C12 labeled FAs of aHSCs transferring to MHCC-97H cells (CellTrace green tracked) (scale bar: 50 µm) and quantifications of mean Red C12 intensity of MHCC-97H cells (Data are represented as mean ± SD, n = 6, at least 50 MHCC-97H cells analyzed per replicate, One-way ANOVA was performed).

Figure S12

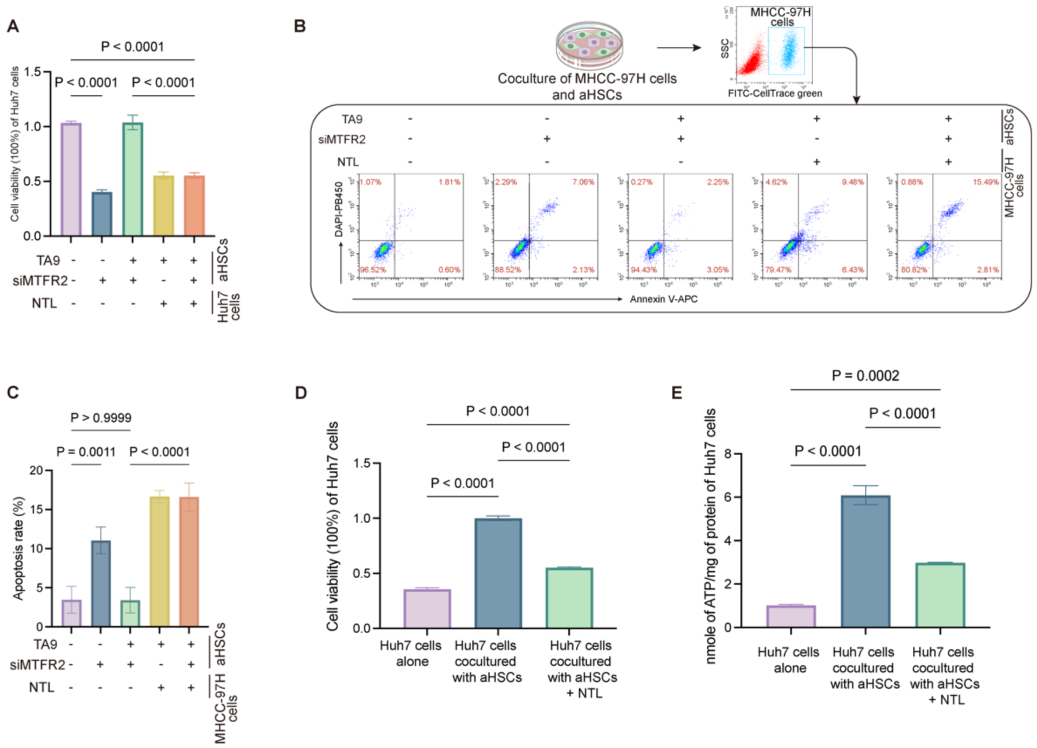

Figure S12. Impact of aHSCs coculture on HCC cell viability, apoptosis and ATP production. A) Analysis of Huh7 cell viability under coculture with various treatment of aHSCs including TA9, siMTFR2, and NTL (Data are represented as mean ± SD, n = 4, One-way ANOVA was performed). B and C) Flow cytometry analysis for apoptosis of CellTrace green-labeled MHCC-97H cells cocultured with aHSCs and Quantitative analysis (C) of total apoptotic cells across experimental groups. (Data are represented as mean ± SD, n = 3, One-way ANOVA was performed). D) Comparison of Huh7 cell viability in three conditions: Huh7 cells alone, Huh7 cells co-cultured with aHSCs, and Huh7 cells co-cultured with aHSCs and treated with NTL (10 μM) (Data are represented as mean ± SD, n = 4, One-way ANOVA was performed). E) Measurement of ATP levels expressed as nmol of ATP/mg of protein in Huh7 cells under different conditions (Data are represented as mean ± SD, n = 3, One-way ANOVA was performed).

Figure S13

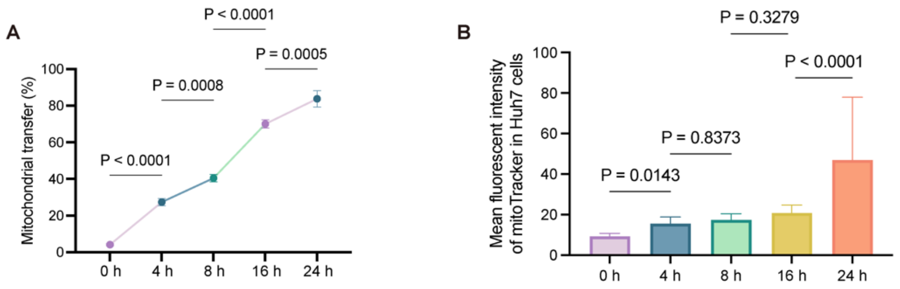

Figure S13. Temporal dynamics of mitochondrial transfer from aHSCs to Huh7 cells. A) Flow cytometric quantification of mitochondrial transfer efficiency. The percentage of dual positive (MitoTracker^{+} CellTrace^{+}) events within the CellTrace^{+} Huh7 cell population (indicative of aHSC-derived mitochondria uptake) was calculated across indicated timepoints (0-24 h), corresponding to Figure 4B (Data are represented as mean ± SD, n = 3, One-way ANOVA was performed). B) Time-course analysis of mitochondrial uptake intensity. Mean fluorescence intensity of MitoTracker in recipient Huh7 cells (CellTrace^{+} population) was plotted against coculture duration, as quantified from the imaging dataset in Figure 4D (Data are represented as mean ± SD, n = 6, One-way ANOVA was performed).

Figure S14

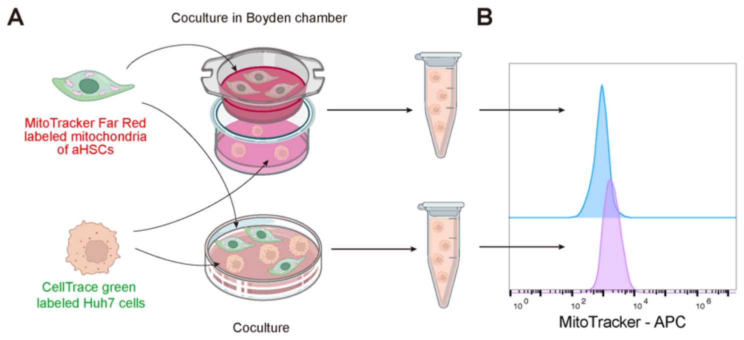

Figure S14. Mitochondrial transfer in two types of coculture models. A) Illustration of the experimental design to evaluate the transfer of mitochondria from aHSCs to Huh7 cells. B) Huh7 cells and aHSCs were loaded with CellTrace green and mitoTracker Far Red, respectively, and employed in the co-culture setup for 24 h. Flow cytometry analysis the fluorescent intensity of mitoTracker Far Red in FITC-positive cells (Huh7 cells).

Figure S15

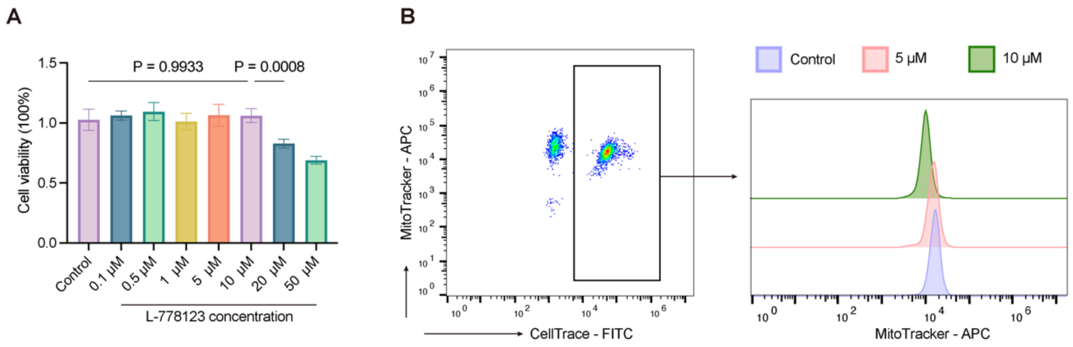

Figure S15. The concentration of L-778123 screening. A) Cell proliferation of aHSCs treating with different concentration of L-778123, and 0.2% DMSO was used as control (Data are represented as mean ± SD, n = 4, One-way ANOVA was performed). B) Flow cytometry evaluated the mitochondria labeled with mitoTracker Far Red of aHSCs transporting to CellTrace green-labeled Huh7 cells. The following experiments were conducted using a concentration of 10 μM.

Figure S16

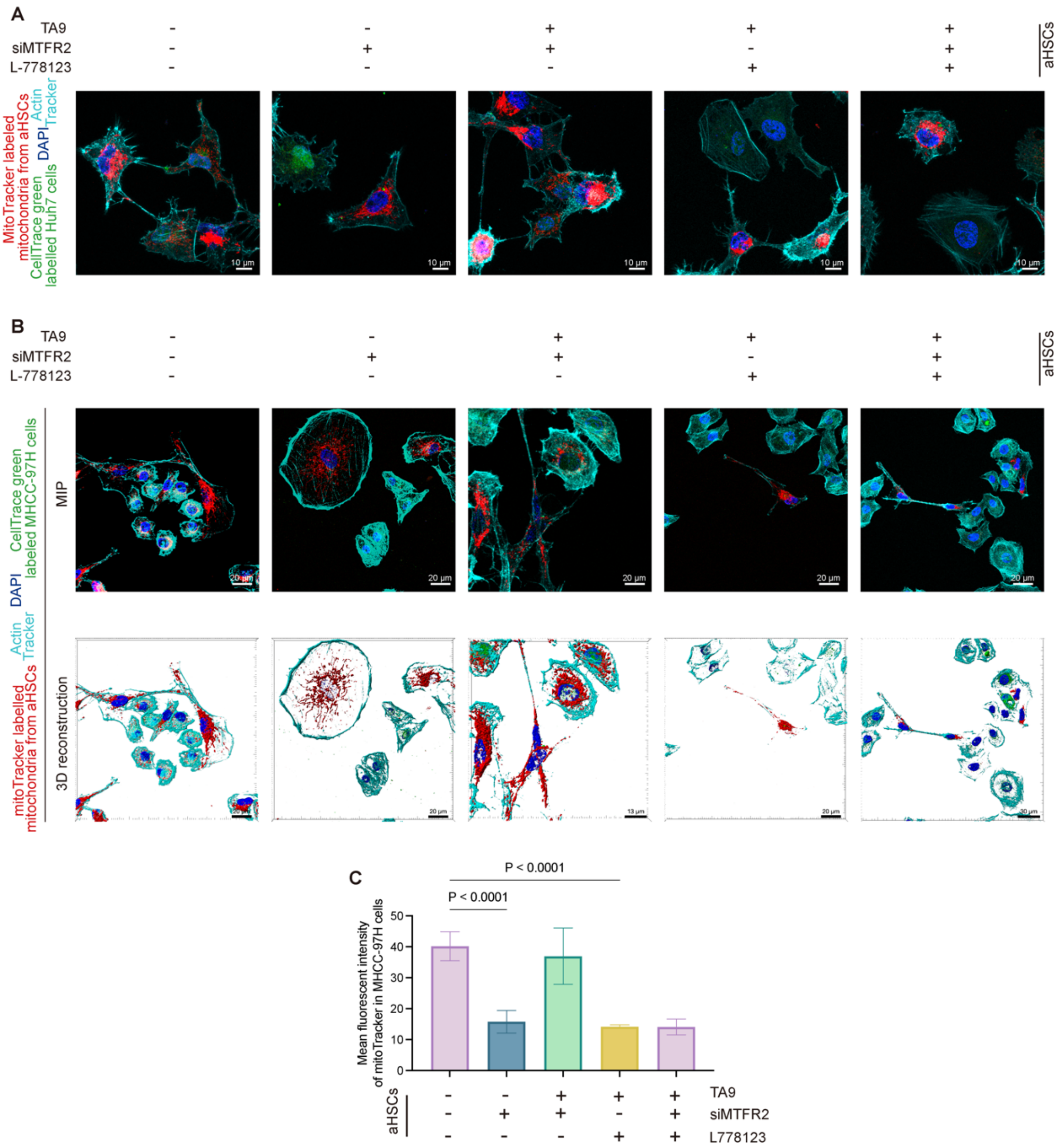

Figure S16. MTFR2-mediated mitochondrial fission facilitates HCC progression through enhanced mitochondrial transfer. A) Maximum intensity projection (MIP) of Z-stack confocal images showing mitochondrial trafficking from mitoTracker-labeled aHSCs (red) to CellTrace-labeled Huh7 cells (green), corresponding to the 3D-rendered data in Figure 4H (scale bar: 10 μm). B and C) MIP images (upper panels) and Imaris-rendered 3D surface plots (downward panels) of MitoTracker-labeled aHSC mitochondria (red) transferred to CellTrace -labeled MHCC-97H cells (green), with quantitative analysis of recipient cell fluorescence intensity (scale bars: 10 μm) (Data are represented as mean ± SD, n = 6, at least 50 MHCC-97H cells analyzed per replicate,

One-way ANOVA was performed).

Figure S17

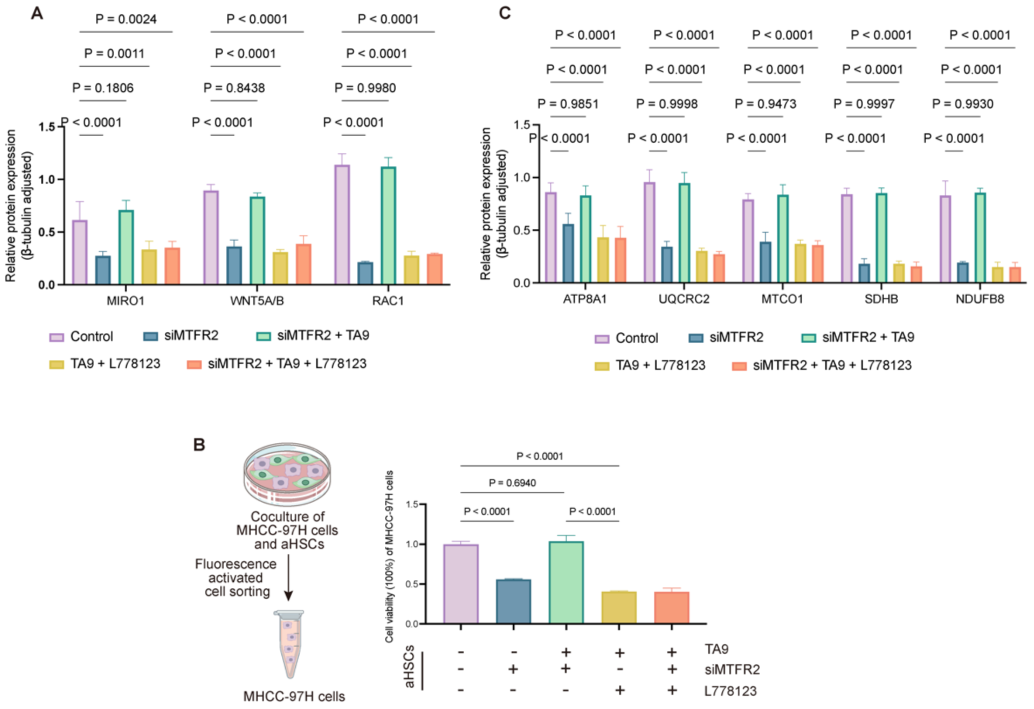

Figure S17. Quantitative protein analysis corresponding to Figure 4J and 4L. A) Densitometric quantification of immunoblotting data shown in Figure 4J (Data are represented as mean ± SD, n = 3, One-way ANOVA was performed). B) The CCK-8 assay evaluated the MHCC-97H cell viability in different conditions (Data are represented as mean ± SD, n = 4, One-way ANOVA was performed). C)Statistical analysis of western blot assays presented in Figure 4L (Data are represented as mean ± SD, n = 3, One-way ANOVA was performed).

Figure S18

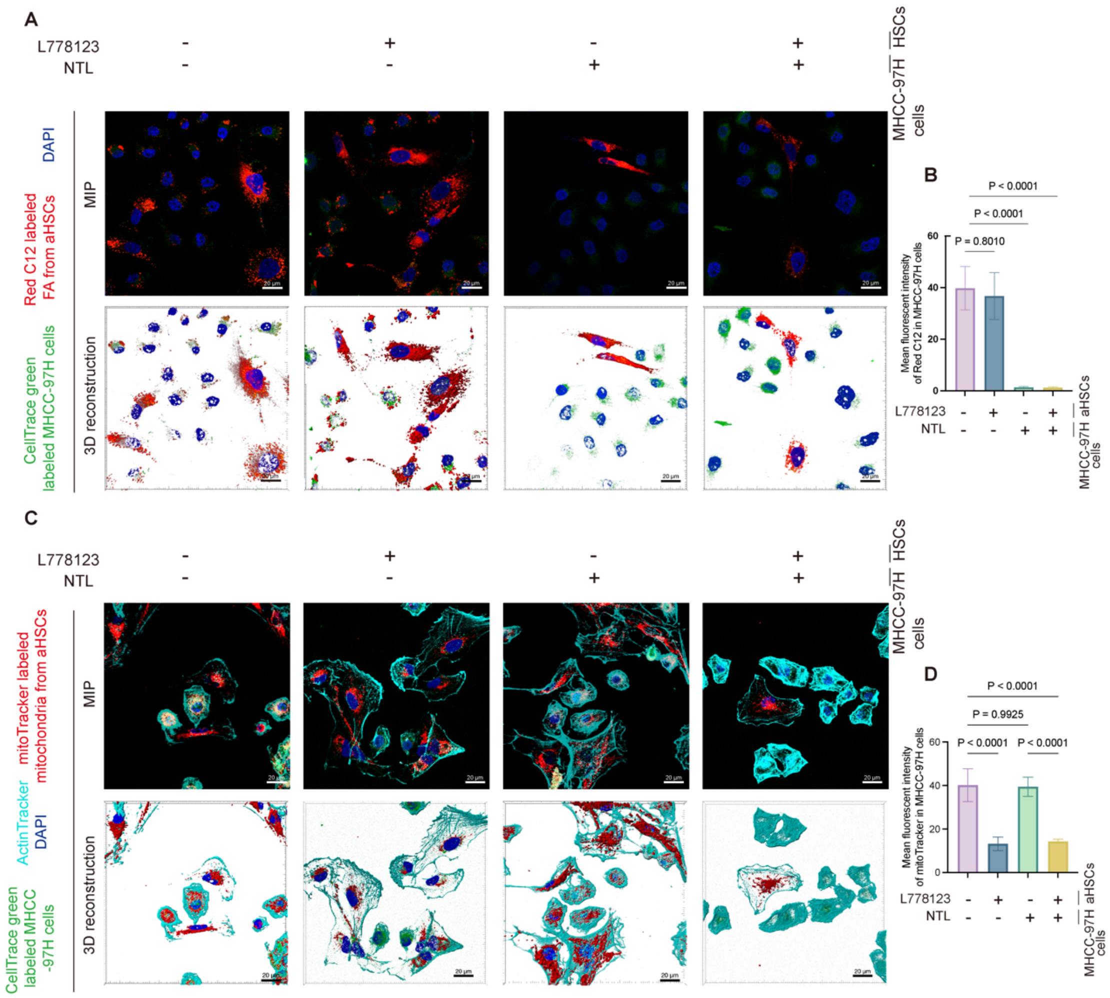

Figure S18. The inhibition of FA and mitochondrial transport from aHSCs to MHCC-97H cells. A and B) Representative confocal MIP (upper panels) and 3D reconstruction images (lower panels) of FA transferred from aHSCs to MHCC-97H cells under various conditions (scale bar: 20 μm) and quantification (B) of mean fluorescent intensity of FA in MHCC-97H cells (Data are represented as mean ± SD, n = 6, at least 50 recipient cells analyzed per replicate, One-way ANOVA was performed). C and D) Mitochondrial transfer dynamics in MHCC-97H cell coculture systems. (C) MIP (upper panels) and 3D-rendered surfaces (lower panels) of MitoTracker-labeled aHSC mitochondria (red) transferred to CellTrace green-labeled MHCC-97H cells (green) under differential conditions. Scale bars: 20 μm. (D) Quantification of mitochondrial transfer efficiency by recipient cell fluorescence intensity (Data are represented as mean ± SD, n = 6, at least 50 recipient cells analyzed per replicate, One-way ANOVA was

performed).

Figure S19

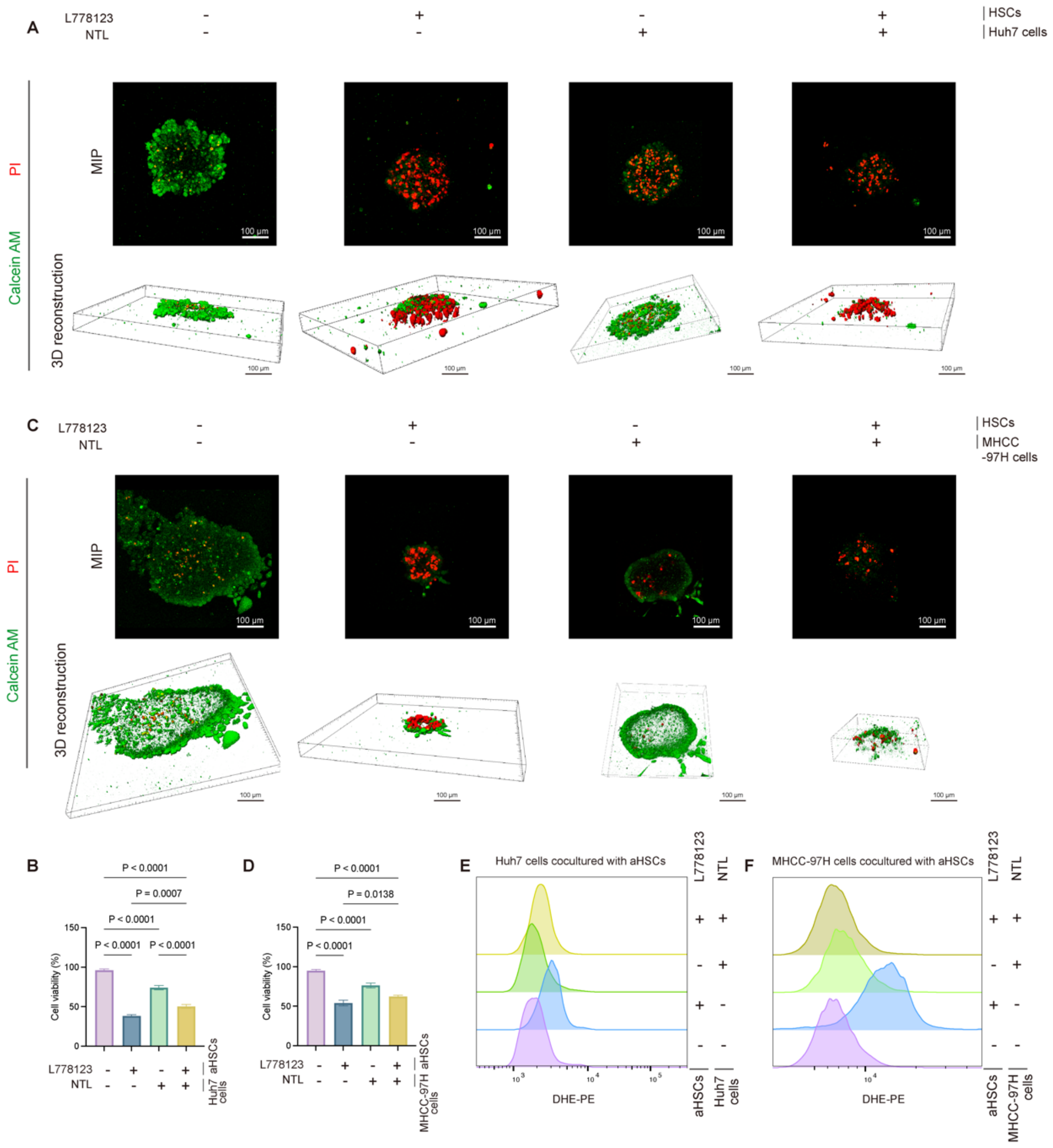

Figure S19. Synergistic roles of FA and mitochondrial transfer accessing in live/dead tumor spheroid using a live (Calcein-AM) and dead (PI) assay, and ROS production evaluated by DHE staining. A and B) Huh7 cell combining aHSCs tumor spheroid images (A, scale bar: 100 μm) and green fluorescence for live cells was quantified and expressed as a percentage of the total fluorescence (red and green) (B) (Data are represented as mean ± SD, n = 3, One-way ANOVA was performed). C and D) MHCC-97H cell combining aHSCs tumor spheroid confocal images (C, scale bar: 100 μm) and quantification fluorescent intensity of green fluorescence for live cells (D) (Data are represented as mean ± SD, n = 3, One-way ANOVA was performed). E, F) Histograms

of DHE fluorescence intensity in Huh7 (E) and MHCC-97H (F) cells (FITC positive) under different conditions.

Figure S20

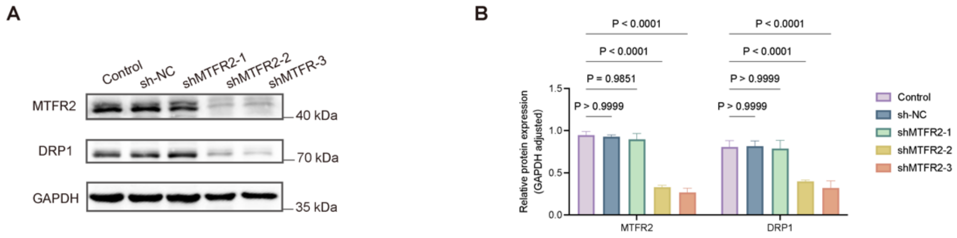

Figure S20. The silencing efficacy of shRNA targeting MTFR2. A, B) Western blot analysis the protein level of MTFR2 and DRP1 and quantification (B) based on GAPDH (Data are represented as mean ± SD, n = 3, Two-way ANOVA was performed).

Figure S21

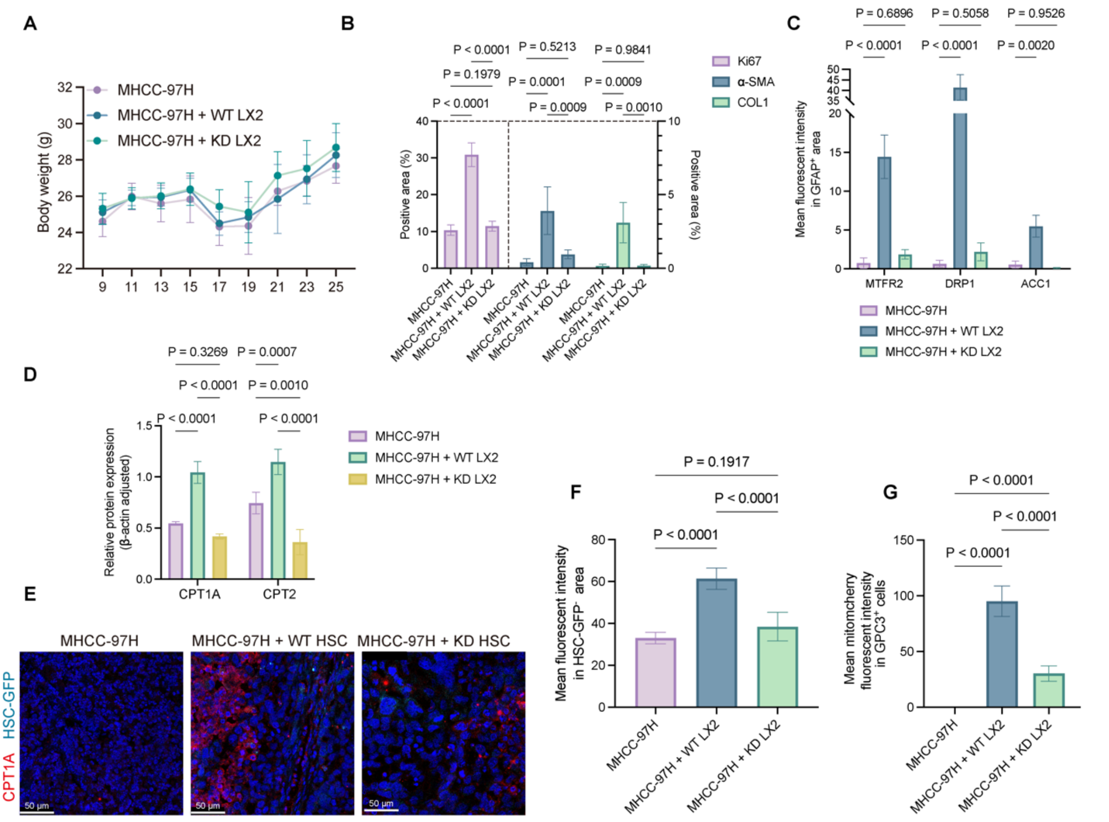

Figure S21. MTFR2 in HSCs promotes tumorigenesis through microenvironmental remodeling. A) body weight changes of the mouse (Data are represented as mean ± SD, n = 6). B) Quantification of IHC-positive area (%) for Ki67, α-SMA, and COL1 in xenograft tumor tissues, as shown in Figure 6E. Data were analyzed using ImageJ software (v 2.14.0) with color deconvolution thresholding and expressed as mean ± SD (n = 6, Two-way ANOVA was performed). C) Mean fluorescent intensity of MTFR2, DRP1, ACC1 in GFAP positive area corresponding to Figure 6F (Data are represented as mean ± SD, n = 6, Two-way ANOVA was performed). D) Quantification of the expression level of CPT1A and CPT2 as shown in Figure 6G (Data are represented as mean ± SD, n = 6, Two-way ANOVA was performed). E and F) Maximum intensity projection (MIP) of Z-stack confocal imaging corresponding to Figure 6H (E, scale bar: 50 μm) and mean fluorescent intensity of CPT1A in HSC-GFP negative area (F, Data are represented as mean ± SD, n = 6, One-way ANOVA was performed). G) Mean mito-mcherry intensity in GPC3 positive cells as shown in Figure 6I (Data are represented

as mean ± SD, n = 6, One-way ANOVA was performed).

Figure S22

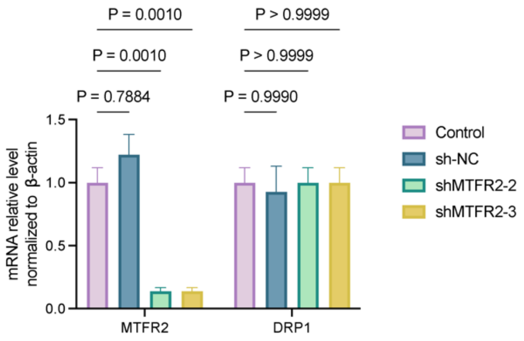

Figure S22. The mRNA relative level of MTFR2 and DRP1 (Data are represented as mean ± SD, n = 3, Two-way ANOVA was performed).

Figure S23

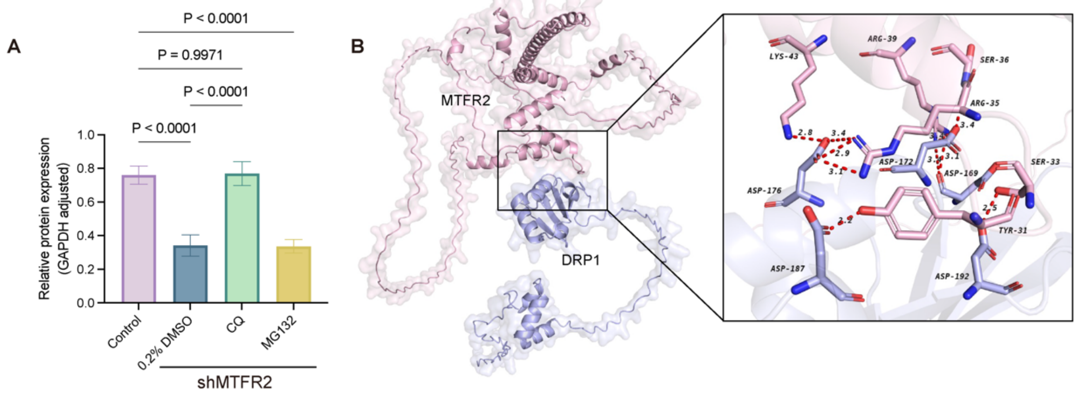

Figure S23. A) Quantification of the protein expression level in Figure 7C, data are represented as mean ± SD, n = 3, One-way ANOVA was performed. B) Predicted binding mode between MTFR2 and DRP1 proteins using AlphaFold3 (https://alphafoldserver.com/). MTFR2 is shown in pink cartoon representation, and DRP1 is shown in blue cartoon mode. Interacting residues are displayed in stick representation, and hydrogen bonds are indicated by red dashed lines.

Figure S24

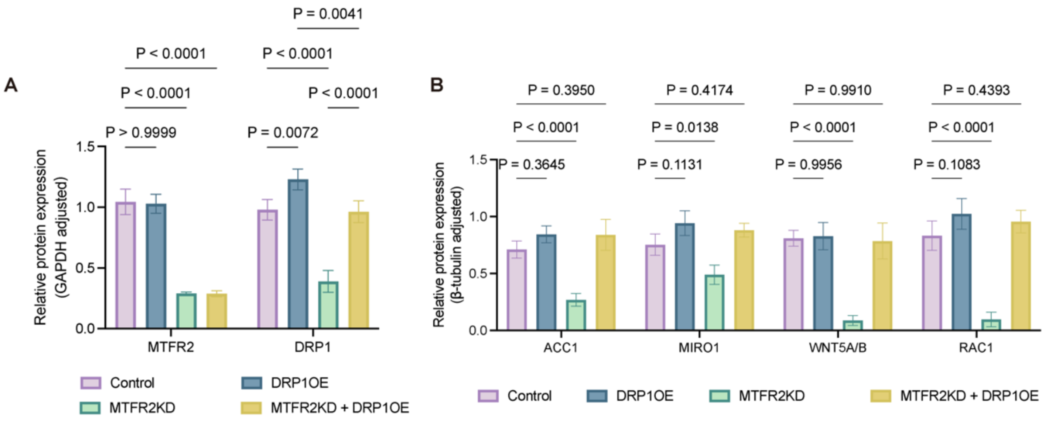

Figure S24. Quantification of the protein expression level in Figure 7E (A) and Figure 7F (B). Data are represented as mean ± SD, n = 3, Two-way ANOVA was performed.

Supporting Tables

Supplementary Table 1. Oligo sequences targeting the MTFR2 gene for small interfering RNA (siRNA).

	Targeting sequence	Sequence (5'-3')
siMTFR2-1	GUACAACCAGGAUCUAAUA	F: GUACAACCAGGAUCUAAUA<br>R: UAUUAGAUCCUGGUUGUAC
siMTFR2-2	GTGGATCTATGGTTCCATCTT	F: GUGGAUCUAUGGUUCCAUCUU<br>R: AAGAUGGAACCAUAGAUCCAC

Supplementary Table 2. Oligo sequences for overexpressing DRP1.

Gene	Sequence (5'-3')
DNM1L	GACTACAAAGACCATGACGGTGATTATAAAGATCATGACATCGACTACAAGGATGACGATGACAAG

Supplementary Table 3. Sequences for short hairpin RNA (shRNA).

	Sequence (5'-3')
shMTFR2-1	GTGGATCTATGGTTCCATCTTCTCGAGAAGATGGAACCATAGATCCAC
shMTFR2-2	CCAAACATGTTGGACGTTCTATTCAAGAGATAGAACGTCCAACATGTTTGG
shMTFR2-3	GCAATTGTGGAAATGCAGGAATTCAAGAGATTCCTGCATTTCCACAATTGC

Supplementary Table 4. The symbols of mitochondrial dynamical genes.

Fission genes	Fusion genes
ARMC10	ARL2
DNM1L	MFN1
FIS1	MFN2
MFF	MIGA1
MIEF1	MIGA2
MIEF2	MTCH2
MTFP1	OMA1
MTFR1	OPA1
MTFR2	PLD6
MUL1	
OMA1	
RAB24	
SLC25A46	
SPIRE1	
STX17	

Supplementary Table 5. Primers for qRT-PCR.

Gene	Sequences (5' to 3')
DNM1L	TTTGGGCGAACCTTAGAA
FAM54A	GCCCATCTGAGTGTGGA
β-actin	GCACCACACCTTCTACAATG
